# Pediatric Outpatient Prescriptions in Countries With Advanced Economies in the 21st Century: A Systematic Review

**DOI:** 10.1001/jamanetworkopen.2022.5964

**Published:** 2022-04-25

**Authors:** Marion Taine, Lucile Offredo, Alain Weill, Rosemary Dray-Spira, Mahmoud Zureik, Martin Chalumeau

**Affiliations:** 1 EPI-PHARE Scientific Interest Group in Epidemiology of Health Products, French National Agency for the Safety of Medecines and Health Products, French National Health Insurance, Saint Denis, France; 2 Obstetrical, Perinatal and Pediatric Epidemiology Research Team, Centre of Research in Epidemiology and Statistics, Université de Paris, National Institute of Health and Medical Research, F-75004, Paris, France; 3 Versailles Saint-Quentin-en-Yvelines University, Versailles, France; 4 Department of General Pediatrics and Pediatric Infectious Diseases, Necker-Enfants Malades Hospital, Assistance Publique-Hôpitaux de Paris, Université de Paris, Paris, France

## Abstract

**Importance:**

An international comparison of pediatric outpatient prescriptions (POPs) is pivotal to investigate inadequate practices at the national scale and guide corrective actions.

**Objective:**

To compare annual POP prevalence among Organisation for Economic Co-operation and Development (OECD) member countries.

**Evidence Review:**

Two independent reviewers systematically searched PubMed, Embase, and institutes of public health or drug agency websites for studies published since 2000 and reporting POP prevalence (expressed as number of patients aged <20 years with ≥1 POP per 1000 pediatric patients per year) in OECD member countries or large geographic areas within them. Risk of bias was assessed for exhaustiveness and representativeness. Prevalence ratios (PRs) were used to compare the highest and lowest POP prevalence among countries overall, by levels of Anatomical Therapeutic Chemical (ATC) classification for the overall pediatric population, and by age group (ie, ages <5-6 vs ≥5-6 years), stratifying on prescription-only drug (POD) status.

**Findings:**

Among 11 studies performed on 3 regional and 8 national medicoadministrative databases in 11 countries, 35 552 550 pediatric patients were included. The overall risk of bias was low (10 studies were representative [90.9%], and the prevalence denominator included nonusers of health care for 9 studies [81.8%]). Prevalence of 1 or more POP per year ranged from 480 to 857 pediatric patients per 1000 in Sweden and France, respectively (PR, 1.8 [95% CI, 1.8-1.8]). Overall, among 8 studies reporting ATC level 1 drugs, Denmark had the lowest POP prevalence (eg, systemic hormonal preparations: 9 pediatric patients per 1000 per year) and France the highest (eg, systemic hormonal preparation: 216 pediatric patients per 1000 per year). Among 8 studies reporting ATC level 2 drugs for PODs, the PR between France and Denmark was 108.2 (95% CI, 108.2-108.2) for systemic corticosteroids and 2.1 (95% CI, 2.1-2.1) for drugs for obstructive airway disease. The PR for antibiotics was 3.4 (95% CI, 3.4-3.4) between New Zealand and Sweden. For pediatric patients aged 5 to 6 years or older, the PR for sex hormones was 2.1 (95% CI, 2.1-2.1) between Denmark and France. Among 7 studies reporting ATC level 5 drugs, the prevalence of the 10 most prevalent PODs was less than 100 pediatric patients per 1000 per year in Scandinavian countries and the Netherlands and less than 300 pediatric patients per 1000 per year in France and New Zealand.

**Conclusions and Relevance:**

This study found large between-country variations in POPs, which may suggest substantial inappropriate prescriptions. The findings may suggest guidance for educational campaigns and regulatory decisions in some OECD member countries.

## Key Points

**Question:**

Among Organisation for Economic Co-operation and Development member countries, is there variation in pediatric outpatient prescriptions?

**Findings:**

This systematic review, based on 11 studies among 35 552 550 pediatric patients performed in 11 countries and regions within them, found geographical disparities in annual pediatric outpatient prescription prevalence. Variation was associated with differences in policies for prescribing drugs available as nonprescription drugs, but wide variations were also found for prescription-only major therapeutic classes, such as systemic corticosteroids and systemic antibiotics.

**Meaning:**

These findings suggest that there may be substantial inappropriate prescriptions in countries with advanced economies.

## Teaser

This systematic review investigates variation in pediatric outpatient prescriptions among 11 Organisation for Economic Co-operation and Development member countries in the 21st century.

## Introduction

The World Health Organization (WHO) defines rational drug prescribing as appropriate medicines in adapted doses for an adequate period of time.^1^ In Organisation for Economic Co-operation and Development (OECD) member countries,^2^ where drugs are reimbursed by health insurance schemes for large parts of the population, irrational drug prescribing may be frequent, especially for outpatient prescriptions.^1,3,4^ The pediatric population, notably the youngest children, should be prioritized for monitoring irrational outpatient drug use because of developmental immaturity and a lower level of evaluation of drugs compared with the adult population, despite the introduction of new regulations.^5^ Indeed, several severe rare or long-term adverse effects were found for drugs used at high rates in pediatrics.^6,7,8,9,10^ Furthermore, encounters between the pediatric population and potential prescribers are frequent in the first years of life because of numerous symptoms and self-limited diseases.^11,12^ Irrational prescribing may vary among countries by primary care physicians’ initial and continuous education,^4^ parental health literacy and attitudes toward drugs,^3,13^ drug regulatory authority policies,^14^ and health system characteristics.^15^ Because epidemiological patterns of the main pediatric diseases are similar in countries with advanced economies,^16^ comparing the prevalence of pediatric outpatient prescriptions (POPs) among these countries may reveal substantial discrepancies regarding inadequate practices at the national scale and may guide corrective actions.^17^

Two studies^18,19^ reported comparisons of POPs in OECD member countries. In 1997, a cohort study^18^ of 12 264 pediatric patients living in 7 European cities found important geographic variations in POPs, mainly for anti-infective agents, with prevalence ratios (PRs) of 2 to 3. In 2008, Sturkenboom et al^18,19^ studied POPs among 675 868 pediatric patients in 3 European countries and also reported high between-country PRs (5 to 20) for antibiotics and systemic corticosteroids. Since these publications, some studies^20,21,22,23^ found an improvement or a worsening of inappropriate POPs over time in some OECD member countries. The only systematic review^24^ of POPs, conducted in 2008, reported great variability in the prevalence of exposure to 1 or more drugs in pediatric patients over the course of a year (from 51% in Denmark to 70% in Greenland). However, that systematic review is more than a decade old, and the comparative evaluation was hampered by heterogeneity among studies. This heterogeneity is mainly associated with differences in information sources (eg, databases of prescription, dispensation, and sales or consumer surveys), indicators (eg, prevalence, share, prescription rate, and package number),^17^ exhaustiveness of the studied population (notably, the inclusion of participants who did not use or slightly used the health care system), and information on nonprescription drugs (NPDs).^25^

We aimed to investigate therapeutic classes and drugs that may be misused and guide corrective actions to improve rational drug prescribing and avoid adverse effects associated with drugs at the population scale. To do so, we systematically evaluated and compared recent annual POP prevalence among OECD member countries while accounting for heterogeneity of drug use–monitoring systems and study designs.

## Methods

### General Methodology

This systematic review was registered at PROSPERO (CRD42021250699) and conducted and reported following international recommendations, including following the Preferred Reporting Items for Systematic Reviews and Meta-analyses (PRISMA) reporting guideline.^26,27^ We systematically searched for reports of the annual prevalence of prescriptions in a pediatric outpatient population (defined as patients aged <20 years) from a medicoadministrative database in an entire OECD member country or large geographic area within it.^2^ We excluded articles focusing on a specific drug or therapeutic class, a short period of time (ie, <1 year), or an age class within the pediatric age group (ie, infants, preschool-age children, school-age children, or adolescents).

We used 2 strategies to search PubMed and Embase databases for articles published between January 1, 2000, and January 1, 2021. Search strategies involved Medical Subject Headings (MESH), the *Emtree* thesaurus, and keywords associated with drug prescription and dispensation, pediatric outpatients, and medicoadministrative databases (eTable 1 in the Supplement). For included studies, we examined reference lists, searched the Science Citation Index and Google Scholar for studies citing the included studies, and examined the studies’ first 50 related articles in PubMed. We also systematically searched institutes of public health or drug agency websites of 37 OECD member countries (eTable 2 in the Supplement).^2^ No language restriction was applied. If data from different studies were from the same country, we selected the most recent study.

Because prescriptions and reimbursements for drugs that are also available as NPDs vary among countries and may have interfered in POP assessment and comparison,^15,28,29,30,31^ we extracted the list of NPDs in all countries for which 1 study was included in the comparison (eTable 3 in the Supplement). Then, we stratified analyses on the prescription-only drug (POD) status (see following sections).

Two authors (M.T. and L.O.) independently screened titles, abstracts, and full texts; extracted data; and assessed risk of bias. A third author (M.C.) was consulted in case of discrepancies.

### Risk of Bias Assessment

We evaluated risk of bias (low, high, or unclear) by adapting tools proposed by Hoy et al^32^ and Munn et al^33^ for prevalence studies based on 2 dimensions (eTable 4 in the Supplement). Internal validity was assessed by the reliability of the data source (ie, prescription vs dispensation database) and exhaustiveness of the population to build the prevalence indicator (ie, populations including nonusers of health care vs populations excluding them). Threat to generalizability was assessed on the selection risk of the population and the use of a tool to improve the representativeness of the study population (ie, census process or random selection).

### Statistical Analysis

We described general characteristics of the included studies and the type of information they reported: levels of Anatomical Therapeutic Chemical (ATC) classification reported (or other classification used), annual overall POP prevalence, and details for age groups within pediatric populations. We described the evaluation of studies’ risk of bias.

We used ATC classification, as recommended by WHO,^17^ to hierarchically classify drugs into anatomical group (first level; eg, nervous system, N), pharmacological and therapeutic subgroup (second level; eg, psychoanaleptic, N06) and chemical substance (fifth level; eg, methylphenidate, N06BA04). Annual POP prevalence was defined as the number of pediatric patients exposed to at least 1 drug divided by the total number of pediatric patients over a calendar year (expressed as the number of pediatric patients with ≥1 prescription per 1000 pediatric patients per year). Thus, if data were missing for higher ATC-level prevalence, we were not able to recalculate them based on lower levels.

We reclassified levels 1 and 2 of the ATC classification into 2 categories. The first included ATC levels 1 and 2 containing only PODs (eg, systemic antibiotics and corticosteroids). The second category was based on the remaining levels 1 and 2 of the ATC containing 1 or more NPDs (eg, nervous system drugs or analgesics that include paracetamol, an NPD that can also be reimbursed if prescribed in France and New Zealand). In this second category, comparisons between POP countries could be hampered by different countries’ policies on prescribing and reimbursement of drugs available as NPDs.^15,28,29,30,31^

We described annual POP prevalence overall and by levels 1 and 2 of the ATC classification, by the 2 categories (ie, containing only PODs vs containing 1 or more NPDs) cited previously, and by age group (ie, ages <5-6 and ≥5-6 years, as suggested by previous articles).^20,22^ We reported the 10 most commonly prescribed PODs (level 5 of the ATC classification) by study.

Prevalence differences (PDs) and PRs were used to compare POPs between countries with the lowest and highest prevalence. Given the large sample sizes for all studies except one,^34^ 95% CIs of POP prevalence was not reported because values with 1 decimal point did not differ from estimates. For the smallest studies and for PD and PR, 95% CIs were reported. Because all statistical comparisons were significant given the number of observations, we arbitrarily defined significant intercountry variation as a PD greater than 20 pediatric patients per 1000 per year and a PR greater than 2. We also reported between-country variation for antiepileptic drugs prescribed for a prototypical disease of this therapeutic class in pediatrics: epilepsy.^35,36^ To limit period bias, we did not compare POP prevalence for data collected during 2009 and before (ie, the median of the studied period). Given the younger age limit of the Dutch study (age <15 years) compared with other studies, we excluded this study for comparisons including adolescent populations. For PODs, we performed sensitivity analyses by comparing studies reporting the same age groups (Table 1).^19,20,22,34,37,38,39,40,41,42,43^ Analyses were conducted from May to June 2021 with the statistical software Excel version 14.2.0 (Microsoft).

**Table 1.** Characteristics of Studies by Study Period

Characteristic	Fernandez-Liz et al,^37^ 2008; Catalonia, Spain	Sturkenboom et al,^19^ 2008; 3 European countries^a^	Zhang et al,^38^ 2013; British Columbia, Canada	Zhong et al,^34^ 2013; Olmsted County, MN, US	Tomlin et al,^20^ 2018; New Zealand	AIFA^39^; Italy	Taine et al,^22^ 2021; France	Sundhedsdata- Styrelsen^40^Denmark	Zorginstituut Nedderland^41^; the Netherlands	NIPH^42^; Norway	Social-Styrelsen^43^; Sweden
Study period, y	2002	2005	2007	2009	2010-2015	2018	2018-2019	2019	2019	2019	2019
Patients, No.	766 398	675 868	855 541	38 558	1 496 026	9 800 000	14 421 749	1 160 384	2 739 819	1 218 965	2 379 242
Databases											
Prescription	No	Yes	No	Yes	No	No	No	No	No	No	No
Dispensation	Yes	No	Yes	No	Yes	Yes	Yes	Yes	Yes	Yes	Yes
Representative	Yes^b^	No	Yes^c^	Yes^c^	Yes	Yes	Yes	Yes	Yes	Yes	Yes
Prevalence denominator includes nonusers of health care	Yes	No	Yes	Yes	No	Yes	Yes	Yes	Yes^b^	Yes	Yes
Any drug prevalence	Yes	No	Yes	No	Yes	Yes	Yes	Yes	No	Yes	Yes
Classification											
ATC 1	No	Yes	No	No	Yes	Yes	Yes	Yes	Yes	Yes	Yes
ATC 2	No	Yes	Yes^d^	Yes^d^	Yes	No	Yes	Yes	Yes	Yes	Yes
ATC 3-4	Yes^d^	No	Yes^d^	Yes^d^	No	No	Yes^d^	Yes	Yes	Yes	Yes
ATC 5	No	No	No	No	Yes	Yes	Yes	Yes	Yes	Yes	Yes
Other than ATC	No	No	No	Yes^e^	No	No	No	No	No	No	No
Age group, y											
<5-6	Yes (0-4 y)	No	Yes (0-5 y)	No	Yes (0-5 y)	Yes (0-5 y)	Yes (0-5 y)	Yes (0-5 y)	Yes (0-4 y)	Yes (0-4 y)	Yes (0-4 y)
≥5-6	No	No	Yes (6-17 y)	No	Yes (6-17 y)	Yes (6-17 y)	Yes (6-17 y)	Yes (6-17 y)	Yes (5-14 y)	Yes (5-19 y)	Yes (5-19 y)
Any age	Yes (0-14 y)	Yes (0-18 y)	Yes (0-17 y)	Yes (0-18 y)	Yes (0-17 y)	Yes (0-17 y)	Yes (0-17 y)	Yes (0-17 y)	NA	Yes (0-19 y)	Yes (0-19 y)

Abbreviations: AIFA, Agenzia Italiana del Farmaco; ATC, Anatomical Therapeutic Chemical; NIPH, Norwegian Institute of Public Health.

^a^ Aggregated data from 3 European countries: Italy (129 487 patients [19.2%]), the Netherlands (101 559 patients [15.0%]), and the United Kingdom (444 822 patients [65.8%]).

^b^ Information on the number of pediatric patients with drug dispensations was available. Prevalence was calculated by dividing these numbers by corresponding census figures.

^c^ Representative of the area but not representative of the country.

^d^ Selected therapeutic classes.

^e^ National Drug File-Reference terminology.

## Results

### Search Results and Included Studies

**Figure 1.**
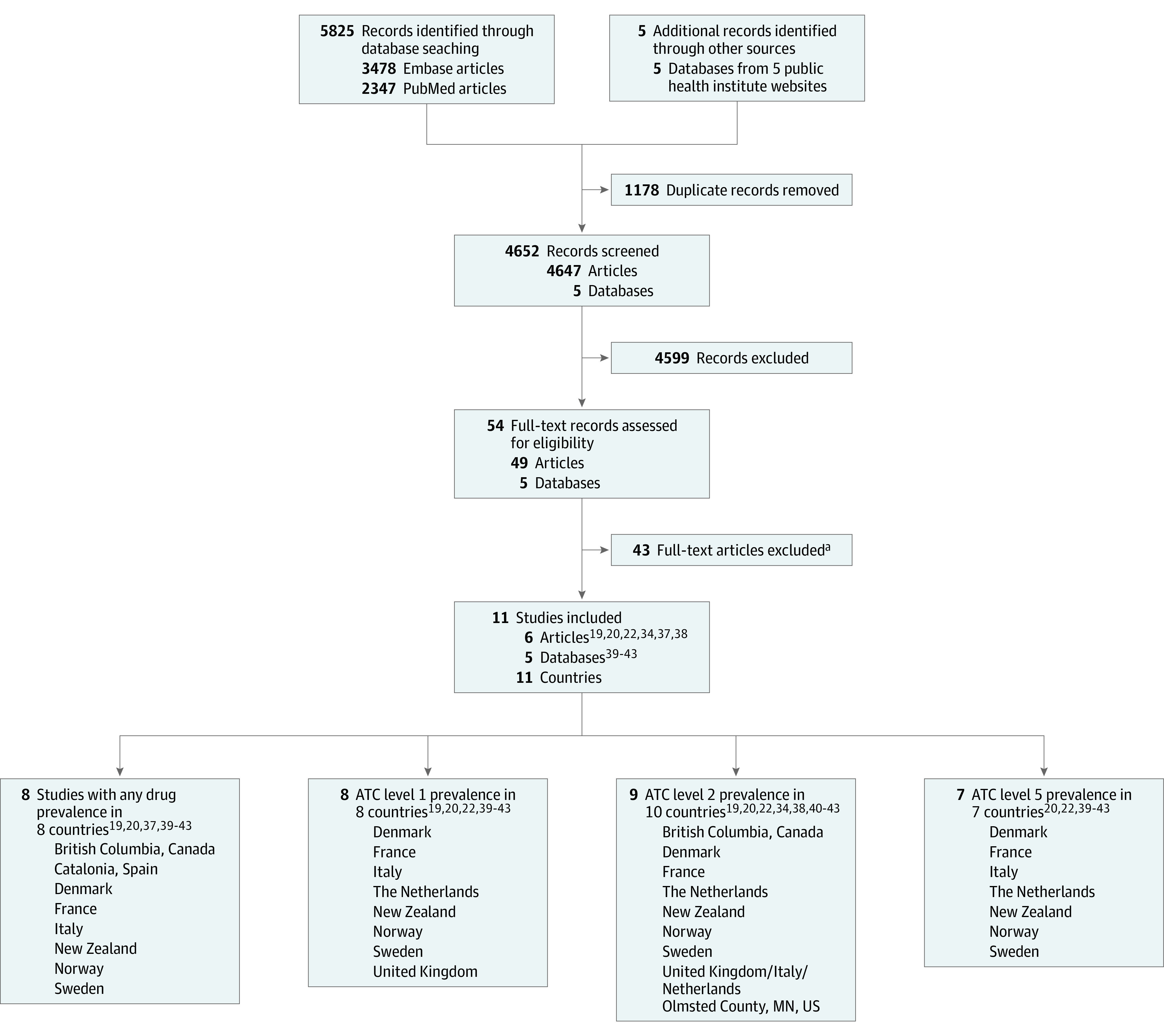
Flowchart of Included Studies ATC indicates Anatomical Therapeutic Chemical. ^a^Includes 2 review articles, 1 article with full text not available, 3 articles with a pediatric population mixed with adult population and indistinguishable, 6 articles with a selected population, 7 articles with selected drugs, 4 articles with over-the-counter drugs included and indistinguishable, 5 articles with indicators other than prevalence (4 with shares and 1 with package number), 7 articles with no annual prevalence data (1 with prevalence for 2 days, 3 with prevalence for 1 month, 1 with prevalence for 6 months, and 2 with prevalence for 4 or 5 years), and 8 articles with data from countries already represented in the study and less recent than those included (2 articles for Sweden, 5 articles for Italy, and 1 article for France).

Among 4647 articles retrieved from the search strategy (Figure 1), 6 articles fulfilled eligibility criteria.^19,20,22,34,37,38^ Websites of institutes of public health or drug agencies provided annual national POP prevalence for 5 countries (eTable 2 in the Supplement).^39,40,41,42,43^ The 11 included studies^19,20,22,34,37,38,39,40,41,42,43^ performed in 11 different countries (Table 1) were based on data for 35 552 550 pediatric patients from North America,^34,38^ Europe,^19,37,39,40,41,42,43^ and New Zealand.^20^ There were 3 regional studies^34,37,38^ and 8 national studies.^19,20,22,39,40,41,42,43^ Data were collected between 2002 and 2019 and after 2010 for 7 studies.^20,22,39,40,41,42,43^ Eight studies^19,20,37,39,40,41,42,43^ reported annual overall POP prevalence, and 8 studies^19,20,22,39,40,41,42,43^ reported annual prevalence by level 1 of ATC classification, 9 studies^19,20,22,34,38,40,41,42,43^ by level 2, and 7 studies^20,22,39,40,41,42,43^ by level 5 (Figure 1 and Table 1). Nine studies reported detailed POPs by age group.^20,22,37,38,39,40,41,42,43^ The overall risk of bias was low; 10 studies^20,22,34,37,38,39,40,41,42,43^ were representative (90.9%), and the prevalence denominator included nonusers of health care for 9 studies (81.8%)^22,34,37,38,39,40,41,42,43^ (Table 1). The most frequent concern was associated with internal validity and use of a proxy of prescriptions in 9 studies based on reimbursed dispensation databases.^20,22,37,38,39,40,41,42,43^

### Between-Country Variation in POPs

**Figure 2.**
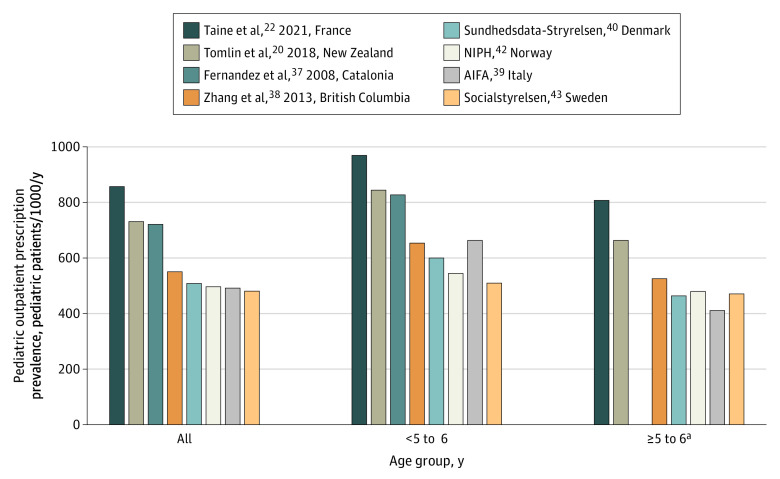
Pediatric Outpatient Prescription Prevalence AIFA indicates Agenzia Italiana del Farmaco; NIPH, Norwegian Institute of Public Health. Prevalence includes nonprescription drugs and is expressed as the frequency of pediatric patients receiving 1 prescription or dispensation or more per 1000 pediatric patients per year. ^a^Pediatric outpatient prescription prevalence for the age group 5 to 6 years or older was not available in the study by Fernandez-Liz et al.^37^

France and New Zealand had the highest annual overall POP prevalence (857 and 731 pediatric patients per 1000 per year, respectively), whereas Scandinavian countries (ranging from 480 pediatric patients per 1000 per year for Sweden to 508 pediatric patients per 1000 per year for Denmark) and Italy (491 pediatric patients per 1000 per year) had the lowest (Figure 2).^20,22,37,38,39,40,42,43^ The PR for France vs Sweden for POPs overall was 1.8 (95% CI, 1.8-1.8). This gradient was almost the same after stratification by age group (ie, ages <5-6 and ≥5-6 years) (Figure 2). The highest prevalence of most levels 1 and 2 of the ATC classification was in France, and the lowest was in Denmark (eg, systemic corticosteroids, 209.9 vs 1.9 pediatric patients per 1000 per year) (Table 2; eTables 5 and 6 in the Supplement).^19,20,22,34,38,40,41,42,43^

**Table 2.** Prevalence of Drug Dispensation or Prescription by Classification^a^

ATC level 2 label	Code	Pediatric patients with dispensations or prescriptions, No./1000 pediatric patients/y									PR (95% CI)^f^	PD (95% CI)^f^
		Sturkenboom et al,^19^ 2008; 3 European countries^b^^,^^c^	Zhang et al,^38^ 2013; British Columbia, Canada^c^	Zhong et al,^34^ 2013; Olmsted County, MN, US^c^	Zorginstituut Nedderland^41^; the Netherlands^d^	Tomlin et al,^20^ 2018; New Zealand	Taine et al,^22^ 2021; France	Sundhedsdata- Styrelsen^40^; Denmark	NIPH^42^; Norway^e^	Social- Styrelsen^43^; Sweden^e^		
Study period	NA	2005	2007	2009	2019	2015	2018-2019	2019	2019	2019	NA	NA
Bile and liver therapy	A05	0.1	NA	NA	0.1	NA	0.5^g^	0.1^h^	0.1	0.2	4.8 (4.7-5.0)	0.4 (0.4-0.4)
Digestive (enzyme)	A09	0.2	NA	NA	0.2	NA	0.2	0.2	0.1^h^	0.2^g^	1.6 (1.4-1.8)	0.1 (0.1-0.1)
Drug used in diabetes	A10	1.5	NA	2.5 (2.0-3.0)	1.4	NA	1.8^h^	2.7	3.3	4.0^g^	2.2 (2.2-2.3)	2.2 (2.1-2.3)
Cardiac therapy	C01	2.2	NA	NA	4.2	NA	5.1	1.7^h^	8.3^g^	5.6	4.9 (4.9-5.0)	6.6 (6.5-6.8)
Antihypertensive	C02	NA	NA	NA	0.3	NA	0.1^h^	0.3	0.5	2.9^g^	32 (31.9-32.0)	2.8 (2.7-2.9)
Diuretic	C03	0.4	NA	1.2 (0.8-1.6)	0.3	NA	0.3	0.3^h^	0.3	0.4^g^	1.4 (1.2-1.5)	0.1 (0.1-0.1)
β-blocking agent	C07	1.2	NA	2.0 (1.6-2.4)	0.8	NA	1.2	1.1^h^	1.5	1.7^g^	1.6 (1.5-1.7)	0.6 (0.5-0.7)
Calcium channel blocker	C08	0.2	NA	0.7 (0.4-1.0)	0.2	NA	0.2	0.2	0.3^g^	0.2^h^	1.7 (1.5-1.9)	0.1 (0.1-0.1)
Renin angiotensin agent	C09	0.3	NA	0.8 (0.5-1.1)	0.5	NA	0.5	0.5^h^	1.2^g^	0.7	2.5 (2.4-2.6)	0.7 (0.6-0.8)
Lipid-modifying agent	C10	0.1	NA	NA	0.3	NA	0.2	0.2^h^	0.7^g^	0.2	4.1 (4.0-4.3)	0.5 (0.5-0.5)
Sex hormone	G03	28.3	24.0	24 (22-25)	7.4^e^	22.0	20.6^h^	42.5	57.2^e^^,^^g^	47.3^e^	2.8 (2.8-2.8)^i^	36.6 (36.2-37.0)^i^
Urological	G04	1.4	NA	NA	1.8	NA	1.7	1.4	1.4^h^	1.8^g^	1.3 (1.2-1.3)	0.4 (0.3-0.5)
Hypothalamic hormone	H01	3.0	NA	2.1 (1.6-2.6)	2.2	NA	4.2^h^	5.2	8.7^g^	5.1	2.1 (2.0-2.1)	4.5 (4.3-4.6)
Corticosteroid (systemic)	H02	22.9	NA	38.9 (37.0-40.8)	5.7	82.0	209.9^g^	1.9^h^	9.9	15.9	108.2 (108.2-108.2)^i^	208.0 (207.5-208.5)^i^
Thyroid therapy	H03	0.9	NA	2.4 (1.9-2.9)	1.2	NA	1.7	1.3^h^	2.5	3.2^g^	2.4 (2.4-2.5)	1.9 (1.8-2.0)
Pancreatic hormone	H04	0.4	NA	NA	0.8	NA	1.5	1.0^h^	1.7^g^	1.4	1.7 (1.6-1.8)	0.7 (0.6-0.8)
Antibiotic (systemic)	J01	270.7	271.0	NA	75.1	480.0^g^	404.8	171.3	142.7	141.2^h^	3.4 (3.4-3.4)^i^	338.7 (337.7-339.6)^i^
Antimycobacterial	J04	0.5	NA	NA	0.2	NA	0.5^g^	0.3	0.2	0.2^h^	18.2 (17.8-18.5)	0.5 (0.5-0.5)
Antineoplastic agent	L01	0.2	NA	NA	0.2	NA	0.5^g^	0.2^h^	0.4	0.3	2.2 (2.1-2.3)	0.3 (0.2-0.3)
Immunosuppressant	L04	0.3	NA	NA	0.8	NA	0.9	0.5^h^	2.1^g^	2.1	4.0 (3.9-4.1)	1.6 (1.5-1.7)
Muscle relaxant	M03	0.2	NA	NA	0.2	NA	0.3^h^	0.6	0.3	1.4^g^	5.7 (5.6-5.7)	1.2 (1.1-1.2)
Antiepileptic	N03	3.5	NA	8.6 (7.7-9.5)	1.8	NA	3.9	3.6^h^	5.1	5.2^g^	1.4 (1.4-1.5)	1.5 (1.4-1.7)
Psycho-analeptic	N06	6.2	26.0	NA	24.2	11.0	7.9^h^	17.9	21.9	36.2^g^	4.6 (4.6-4.6)^i^	28.3 (28.0-28.5)^i^
Antiprotozoal	P01	1.9	NA	NA	2.4	NA	3.0^h^	5.3	5.4^g^	5.0	1.7 (1.7-1.8)	2.3 (2.2-2.4)
Drug for obstructive airway disease	R03	100.9	81.0	101.7 (98.7-104.7)	68.9	131.0	143.2^g^	67.3^h^	70.4	80.6	2.1 (2.1-2.1)^i^	76.0 (75.5-76.5)^i^

Abbreviations: ATC, Anatomical Therapeutic Chemical; NA, not available; NIPH, Norwegian Institute of Public Health; PD, prevalence difference; PR, prevalence ratio.

^a^ 95% CIs of prevalence numbers were not reported given the large sample sizes of different studies, except for the study in Olmsted County, Minnesota, US.

^b^ Aggregated data from 3 European countries: Italy (129 487 of 675 868 patients [19.2%]), the Netherlands (101 559 of 675 868 patients [15.0%]), and the United Kingdom (444 822 of 675 868 patients [65.8%]).

^c^ Data are displayed for information purposes but are not included in the comparison because of their age (ie, 2009 or older).

^d^ Data are displayed for information purposes but are not included because of the younger age of the Dutch pediatric population (ie, ages <15 years).

^e^ Norway and Sweden include a pediatric population aged less than 20 years.

^f^ PR and PD are given between the countries with the highest and lowest prevalence of level 2 of the ATC classification.

^g^ Highest prevalence.

^h^ Lowest prevalence.

^i^ PR greater than 2 and PD 20 or more pediatric patients per 1000 per year.

Among 8 studies reporting level 2 of the ATC classification for only PODs, the PR between France and Denmark was 108.2 (95% CI, 108.2-108.2) for systemic corticosteroids and 2.1 (95% CI, 2.1-2.1) obstructive airway disease drugs (Table 2). The PR for antibiotics was 3.4 (95% CI, 3.4-3.4) between New Zealand and Sweden (Table 2). Sensitivity analyses among studies with the same age group did not have different outcomes, except for antibiotics, which had a PR of 2.8 (95% CI, 2.8-2.8) between New Zealand and Denmark in sensitivity analysis (eTable 7 in the Supplement). In these sensitivity analyses, the PRs for antiepileptics were 1.4 (95% CI, 1.4-1.5) between Sweden and France, 1.1 (95% CI, 1.1-1.1) between France and Denmark, and 1.0 (95% CI, 1.0-1.0) between Sweden and Norway (Table 2; eTable 7 in the Supplement).

Variations were observed by age group. POP prevalence was higher among pediatric patients aged less than 5 to 6 years compared with pediatric patients aged 5 to 6 years or older for all countries (Figure 2). The PR for systemic corticosteroids was higher for children younger than 5 to 6 years compared with older pediatric patents in main and sensitivity analyses (655.2 [95% CI, 655.1-655.4] vs 60.9 [95% CI, 60.8- 60.9]) (eTables 8-11 in the Supplement). For pediatric patients aged 5 to 6 years or older, the PR between Sweden and France was 4.4 (95% CI, 4.4-4.4) for psychoanaleptic drugs, and the PR between Norway and France was 2.5 (95% CI, 2.5-2.5) for sex hormones (eTable 10 in the Supplement). In sensitivity analyses, PRs between Denmark and France were 2.3 (95% CI, 2.3-2.3) for psychoanaleptics and 2.1 (95% CI, 2.1-2.1) for sex hormones (eTable 11 in the Supplement).

**Figure 3.**
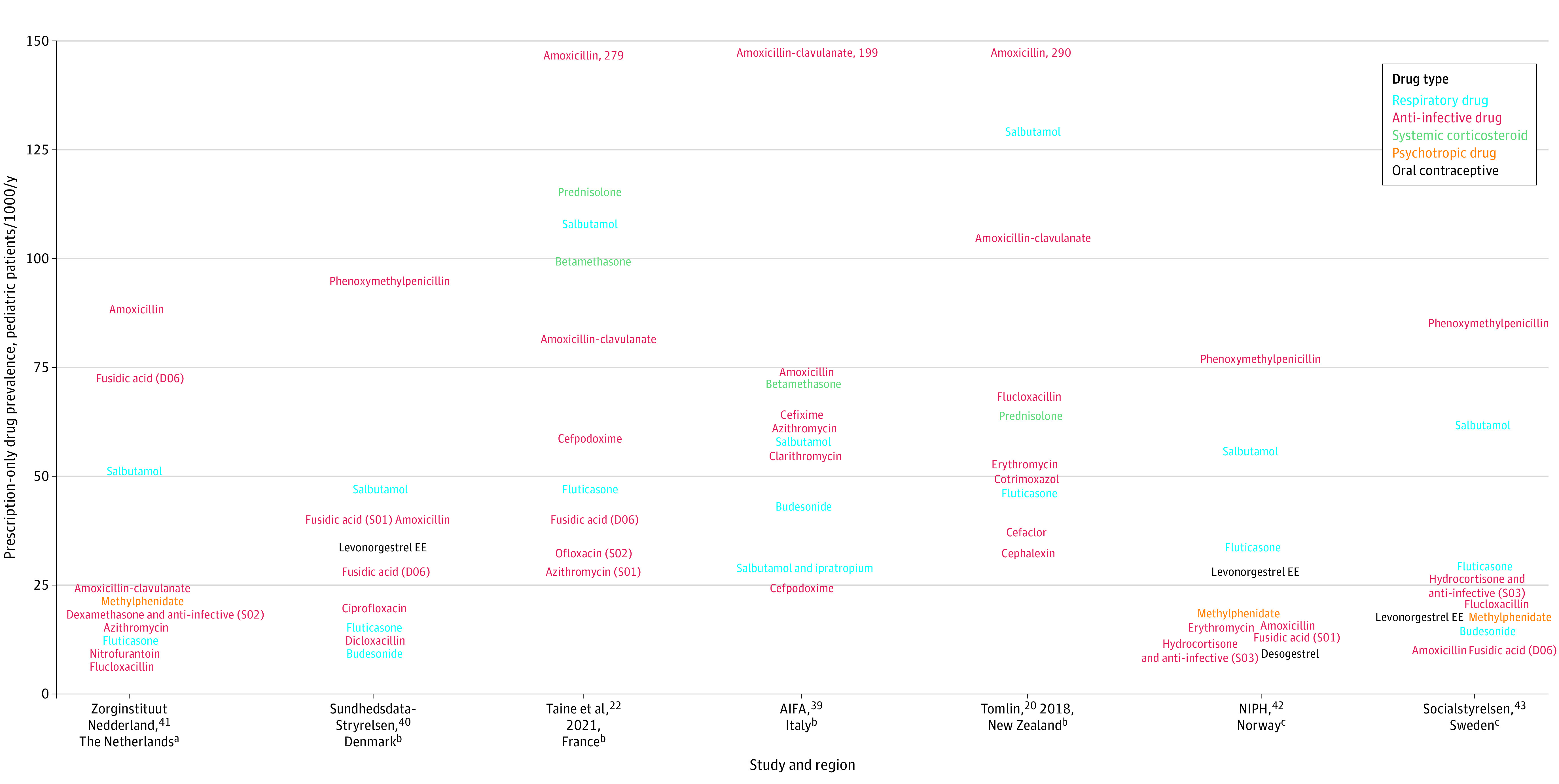
Prevalence of 10 Most Commonly Prescribed Prescription-Only Drugs by Country or Region Prevalence is expressed as frequency of pediatric patients receiving 1 prescription or more per 1000 pediatric patients per year. AIFA indicates Agenzia Italiana del Farmaco; D06, antibiotics and chemotherapeutics for dermatological use; NIPH, Norwegian Institute of Public Health; levonorgestrel EE, levonorgestrel ethinyl estradiol; S01, ophthalmologicals; S02, otologicals; S03, ophthalmological and otological preparations. ^a^Pediatric population aged less than 15 years. ^b^Pediatric population aged less than 18 years. ^c^Pediatric population aged less than 20 years.

Among the 10 most prevalent PODs by country (Figure 3),^20,22,39,40,41,42,43^ the POD with the highest annual prevalence was always an antibiotic: amoxicillin in New Zealand (290 pediatric patients per 1000 per year), France (279 pediatric patients per 1000 per year), and the Netherlands (89 pediatric patients per 1000 per year); amoxicillin-clavulanate in Italy (199 pediatric patients per 1000 per year); and phenoxymethylpenicillin in Denmark (96 pediatric patients per 1000 per year), Sweden (86 pediatric patients per 1000 per year), and Norway (77 pediatric patients per 1000 per year). The most prevalent drugs were antibiotics (7.7-290.0 pediatric patients per 1000 per year) and antiasthmatic drugs (12.1-130.0 pediatric patients per 1000 per year). The remaining drugs were systemic corticosteroids, particularly frequently prescribed in France (prednisolone: 116 pediatric patients per 1000 per year; betamethasone: 100 pediatric patients per 1000 per year), Italy (betamethasone: 75 pediatric patients per 1000 per year), and New Zealand (prednisolone: 65 pediatric patients per 1000 per year), and psychoanaleptic drugs (methylphenidate: from 17-22 pediatric patients per 1000 per year) and contraception drugs (levonorgestrel ethinylestradiol: from 18-35 pediatric patients per 1000 per year), particularly frequently prescribed in Scandinavian countries and the Netherlands. In Scandinavian countries and the Netherlands, the prevalence of the most prevalent PODs was less than 100 pediatric patients per 1000 per year. In France and New Zealand, the prevalence was 30 to nearly 300 pediatric patients per 1000 per year for the most prevalent PODs (Figure 3).

For level 2 drugs of the ATC classification, mixing PODs and drugs available as NPDs, the prevalence of POPs varied widely among countries (eTable 5 in the Supplement). For example, we found the largest differences in POPs, including drugs available as NPDs, when comparing the prevalence for France or New Zealand with that for Denmark (eg, the PDs for analgesics, nasal preparations, and vitamins were 629.2 pediatric patients per 1000 per year [95% CI, 628.9-629.6 pediatric patients per 1000 per year], 294.5 pediatric patients per 1000 per year [95% CI, 294.1-294.9 pediatric patients per 1000 per year], and 303.4 pediatric patients per 1000 per year [95% CI, 303.2-303.6 pediatric patients per 1000 per year], respectively). Similar variations were observed for level 1 drugs of the ATC classification that mixed PODs and drugs available as NPDs (nervous system: PD, 663 pediatric patients per 1000 per year [95% CI, 662.8-663.2 pediatric patients per 1000 per year]; alimentary tract and metabolic system: PD, 477 pediatric patients per 1000 per year [95% CI, 476.6-477.4 pediatric patients per 1000 per year]; respiratory system: PD, 378 pediatric patients per 1000 per year [95% CI, 377.4-378.6 pediatric patients per 1000 per year]) (eTable 6 in the Supplement).

## Discussion

### Main Results

This systematic review found marked geographical disparities in annual POP prevalence among OECD member countries and regions within them. An important part of the variation was associated with differences in policies for prescribing drugs available as NPDs, but wide variations also concerned prescription-only major therapeutic classes, such as systemic corticosteroids, systemic antibiotics, psychoanaleptic drugs, oral contraceptives, and antiasthmatic drugs. These large between-country variations were not likely associated with epidemiological variations in diseases,^16^ and they may instead be associated with different practices and inappropriate overprescribing or underprescribing. These overprescriptions particularly concerned the most immature pediatric age group, given that children aged less than 5 to 6 years old had the highest prevalence of POPs regardless of geographic area.

### Interpretation

This systematic review found that POP prevalence was considerably higher in France and New Zealand than in Italy, the Netherlands, Scandinavian countries, and British Columbia, Canada. These wide variations in POP prevalence may be associated with the heterogeneous structure of health care systems.^44^ The facilitated access to medical care,^44^ high level of drug reimbursement for pediatric populations,^44^ and positive attitudes toward drugs held by physicians and the public^45,46^ may be associated with these patterns of drug prescribing in France and New Zealand. In particular, the prescribing pattern of drugs also available as NPDs in France^25^ contrasts with that in other countries with similar economies that reimburse these drugs only under certain conditions^30^ or not at all if their efficacy was not based on evidence.^15^

This study also found important between-country variations in POPs for systemic corticosteroids. The prevalence of POPs for systemic corticosteroids 108-fold higher in France than in Denmark. This therapeutic class has potential severe adverse effects, such as increased risk of infections^47^ and metabolic disturbances with cumulative exposure to a few courses.^48^ Several hypotheses may explain such gaps between France and other countries. The first difference may be cultural, with physicians in France having a positive attitude toward this therapeutic class,^49^ in contrast to physicians in Scandinavian countries.^50,51^ Furthermore, the management of some diseases may differ among countries. For instance, for otitis media with effusion, Danish recommendations suggest rapid recourse to surgery,^52^ while US guidelines^53^ recommend against use of corticosteroids and French guidelines suggest treatment with systemic or nasal corticosteroids to relieve symptoms while waiting for spontaneous improvement or surgical treatment.^54^ Additionally, a short course of systemic corticosteroids is indicated by the Global Initiative for Asthma as a rescue medication for severe exacerbation of asthma.^55^ The control of asthma may be less optimal among the French pediatric population compared with other countries with lower rates of hospital admissions.^56,57,58^ Additionally, short courses of systemic corticosteroids are often misused to treat moderate and even mild exacerbations and symptoms of asthma in some Western countries.^59,60,61,62^

Between-country POP variations for antibiotics were also substantial. In our study, New Zealand pediatric patients were 2.8-fold and 3.4-fold more likely to receive a prescription for systemic antibiotics than were Danish or Swedish pediatric patients, respectively. Antibiotics are mainly inappropriately prescribed for viral infections in OECD member countries,^46,63,64,65^ and their overuse is associated with bacterial resistance.^63^ Our results suggest the need to accelerate education and regulations regarding the appropriate use of antibiotics in New Zealand.^66^

Another notable between-country POP variation concerned psychoanaleptic drugs. Older pediatric patients in Denmark and Sweden were 2.3-fold and 4.4-fold, respectively, more likely to be prescribed psychoanaleptics than their French peers. For this therapeutic class, 2 molecules are mainly used in pediatrics: fluoxetine (a serotonin reuptake inhibitor)^67^ and methylphenidate (a centrally acting psychostimulant), prescribed for depressive syndrome and attention-deficit/hyperactivity disorder, respectively.^68^ Two studies^68,69^ found a striking increase in use of these 2 drugs during the last 2 decades in Sweden, reaching levels higher than those in most other countries, including neighboring countries, such as Norway and Denmark. The US study^34^ included in this systematic review did not report annual prevalence of POPs for psychoanaleptic drugs, although the US is among the countries with the highest prevalence of POPs for this therapeutic class.^13,70^ These high POP levels may be associated with physician and public attitudes toward psychotropic drugs in Sweden and the US and the facilitated access to psychiatric services for the pediatric population in Sweden.^13^ Conversely, the reluctance to prescribe drugs with a partially known long-term safety profile^71^ and the place of nonpharmaceutical therapeutic approaches^72^ may be associated with the low prevalence of psychotropic drug prescription in other countries.

Another between-country POP discrepancy concerned oral contraceptives. Danish pediatric patients aged 6 years and older were 2.1-fold more likely to be prescribed sex hormones than their French peers. The mean age of initiation of oral contraceptives is between 18 and 20 years in many European countries,^73^ so comparison between French and Norwegian pediatric patients was not possible in our systematic review; the Norwegian population studied included individuals aged 18 to 19 years, unlike the French population. Nevertheless, this finding suggests that female adolescents in Denmark start using oral contraceptives earlier than their French peers.^74,75^ In Scandinavian countries, several factors are positively associated with oral contraceptive use; these include family planning facilities, social policies, and sociodemographic characteristics.^75^ These factors may vary in other OECD member countries.

This systematic review identified another important between-country difference in POPs concerning antiasthmatic drugs. This difference may be associated with the lack of international standardization for the diagnosis and treatment of asthma, especially for children aged younger than 6 years.^76^

### Strengths and Limitations

This study updated a previous systematic review^24^ on annual POP prevalence with 11 new studies. It also allowed for a more in-depth comparison of international prevalence of POPs than previously by comparing therapeutic classes and the most frequently prescribed active substances by country. To improve the quality of comparisons, we stratified by therapeutic classes that included mainly PODs and thus identified irrational prescribing associated with some of these classes. In line with our hypothesis that the overall similar epidemiological patterns of major pediatric diseases in the OECD member countries would be associated with similar prevalence of PODs, we observed that antiepileptics, mainly prescribed for epilepsy,^35,36^ varied little among OECD member countries.

This study has several limitations. First, many OECD member countries did not report annual prevalence of dispensations and prescriptions, including countries with potentially extreme high or low POPs. This reduced the magnitude of variations found in our systematic review. Additionally, among many potential intervals to study POP prevalence (eg, year, month, or week), we selected the annual indicator to allow for homogeneity in comparisons for a period. Thus, we restricted our review to medicoadministrative database studies to capture sporadic users exhaustively, which is not possible in studies for which the prevalence is estimated for a short period.^77^ An implication of this selection process was the exclusion of several studies based on different indicators in North America, Europe, and Asia.^21,78,79^ Of note, North American studies^34,37,38^ included in this systematic review were more than a decade old, so we excluded them from comparisons. Second, we did not investigate POP prevalence for level 3 or 4 drugs of the ATC classification. Third, POP prevalence comparisons were likely biased given the large study period, but we tried to limit this bias by not including data collected before 2010 in comparisons. Additionally, most databases included dispensations and likely underestimated POP prevalence because some prescriptions may not be dispensed if parents or adolescents do not pick up the prescribed drugs from the pharmacy. However, this homogeneity of databases was also a strength for comparisons. Fourth, these administrative databases did not collect information on indications for drug prescriptions, which precludes analysis of the appropriateness of drug prescriptions. Fifth, we compared maximal variations between countries to investigate extreme prescription attitudes. We did this because detecting variations by comparison to an optimal level of POP prevalence by country is rarely possible given that target optimal levels are not available. Furthermore, we did not calculate pooled POPs in a meta-analysis, which would have been difficult to interpret. Sixth, because reported age groups were not exactly concordant, some comparisons should be interpreted with caution, particularly for adolescents. To limit this bias, we excluded the Dutch study^41^ from the comparison analysis and performed sensitivity analyses between studies with the same age group. Indeed, comparisons between POPs observed in studies performed in Sweden and Norway, in which the pediatric population was defined as younger than age 20 years, may have overestimated variations for POPs more commonly prescribed among young adults (eg, contraception drugs, drugs used in diabetes, thyroid therapy, and psychoanaleptics) compared with studies in countries in which the upper limit was 18 years, for example. However, sensitivity analyses had similar results as most of our main findings but with attenuated POP prevalence variations for antibiotics and psychoanaleptics. This limitation suggests a lack of harmonization in reporting of POPs among pediatric age groups and a need for consensual reporting guidelines to standardize reports and allow more robust comparisons. Seventh, we compared data from published and nonpublished studies (ie, websites) that were not externally peer reviewed. However, these websites provided complete data and criteria to evaluate risk of bias.

## Conclusions

This study found considerable international variation in POP prevalence that may be associated with the structure of health care systems, public attitudes toward drugs, NPD prescription by physicians, and national guidelines. The magnitude of variations in POPs for antibiotics, systemic corticosteroids, psychoanaleptic drugs, oral contraceptives, and antiasthmatic drugs suggests substantial inappropriate overprescription and underprescription depending on geographic area and therapeutic class. Our findings suggest that factors associated with POPs among these drugs with potential severe adverse effects deserve to be further explored to guide educational campaigns and regulatory decisions in some OECD member countries.

## Notes

Supplement. **eTable 1.** Search Equations **eTable 2.** Description of Criteria Used for Extracting Annual Prevalence **eTable 3.** Anatomical Therapeutic Chemical Level 2 Classes With and Without Nonprescription Drugs **eTable 4.** Risk of Bias Items Assessed **eTable 5.** Prevalence of Drug Dispensation and Prescription by Anatomical Therapeutic Chemical Level 2 **eTable 6.** Prevalence of Drug Dispensation and Prescription by Anatomical Therapeutic Chemical Level 1 **eTable 7.** Prevalence of Drug Dispensation and Prescription by Anatomical Therapeutic Chemical Level 2 in Same Age Group Studies **eTable 8.** Prevalence of Drug Dispensation and Prescription by Anatomical Therapeutic Chemical Level 2 Among Children Aged Less Than 5 to 6 Years **eTable 9.** Prevalence of Drug Dispensation and Prescription by Anatomical Therapeutic Chemical Level 2 Among Children Aged Less Than 5 to 6 Years in Same Age Group Studies **eTable 10.** Prevalence of Drug Dispensation and Prescription by Anatomical Therapeutic Chemical Level 2 Among Pediatric Patients Aged 5 to 6 Years or Older **eTable 11.** Prevalence of Drug Dispensation and Prescription by Anatomical Therapeutic Chemical Level 2 Among Pediatric Patients Aged 5 to 6 Years or Older in Same Age Group Studies
