# Supplementary material for: Pediatric Outpatient Prescriptions in Countries With Advanced Economies in the 21st Century: A Systematic Review
Source: JAMA Netw Open. 2022 Apr 25;5(4):e225964. doi: 10.1001/jamanetworkopen.2022.5964 (PMC9039774; doi:10.1001/jamanetworkopen.2022.5964)
Supplement: Supplement. — eTable 1. Search Equations eTable 2. Description of Criteria Used for Extracting Annual Prevalence eTable 3. Anatomical Therapeutic Chemical Level 2 Classes With and Without Nonprescription Drugs eTable 4. Risk of Bias Items Assessed eTable 5. Prevalence of Drug Dispensation and Prescription by Anatomical Therapeutic Chemical Level 2 eTable 6. Prevalence of Drug Dispensation and Prescription by Anatomical Therapeutic Chemical Level 1 eTable 7. Prevalence of Drug Dispensation and Prescription by Anatomical Therapeutic Chemical Level 2 in Same Age Group Studies eTable 8. Prevalence of Drug Dispensation and Prescription by Anatomical Therapeutic Chemical Level 2 Among Children Aged Less Than 5 to 6 Years eTable 9. Prevalence of Drug Dispensation and Prescription by Anatomical Therapeutic Chemical Level 2 Among Children Aged Less Than 5 to 6 Years in Same Age Group Studies eTable 10. Prevalence of Drug Dispensation and Prescription by Anatomical Therapeutic Chemical Level 2 Among Pediatric Patients Aged 5 to 6 Years or Older eTable 11. Prevalence of Drug Dispensation and Prescription by Anatomical Therapeutic Chemical Level 2 Among Pediatric Patients Aged 5 to 6 Years or Older in Same Age Group Studies [file jamanetwopen-e225964-s001.pdf]

Supplemental Online Content

Taine M, Offredo L, Weill A, Dray-Spira R, Zureik M, Chalumeau M. Pediatric outpatient prescriptions in countries with advanced economies in the 21st century: a systematic review. *JAMA Netw Open.* 2022;5(4):e225964. doi:10.1001/jamanetworkopen.2022.5964

eTable 1. Search Equations

eTable 2. Description of Criteria Used for Extracting Annual Prevalence

eTable 3. Anatomical Therapeutic Chemical Level 2 Classes With and Without Nonprescription Drugs

eTable 4. Risk of Bias Items Assessed

eTable 5. Prevalence of Drug Dispensation and Prescription by Anatomical Therapeutic Chemical Level 2

eTable 6. Prevalence of Drug Dispensation and Prescription by Anatomical Therapeutic Chemical Level 1

eTable 7. Prevalence of Drug Dispensation and Prescription by Anatomical Therapeutic Chemical Level 2 in Same Age Group Studies

eTable 8. Prevalence of Drug Dispensation and Prescription by Anatomical Therapeutic Chemical Level 2 Among Children Aged Less Than 5 to 6 Years

eTable 9. Prevalence of Drug Dispensation and Prescription by Anatomical Therapeutic Chemical Level 2 Among Children Aged Less Than 5 to 6 Years in Same Age Group Studies

eTable 10. Prevalence of Drug Dispensation and Prescription by Anatomical Therapeutic Chemical Level 2 Among Pediatric Patients Aged 5 to 6 Years or Older

eTable 11. Prevalence of Drug Dispensation and Prescription by Anatomical Therapeutic Chemical Level 2 Among Pediatric Patients Aged 5 to 6 Years or Older in Same Age Group Studies

This supplemental material has been provided by the authors to give readers additional information about their work.

© 2022 Taine M et al. *JAMA Network Open.*

eTable 1. Search Equations

Pubmed

#1. ((((((((outpatients [MeSH Terms]) OR outpatient [Title/Abstract]) OR population-based [Title/Abstract]) OR noninstitutionalized [Title/Abstract]) OR pharmacy [Title/Abstract]) OR pharmacies [Title/Abstract]) OR representative [Title/Abstract]) OR nation* [Title/Abstract]) OR survey [Title/Abstract])

#2. (((((((((paediatric [Title/Abstract]) OR pediatric [Title/Abstract]) OR adolescen* [Title/Abstract]) OR infan* [Title/Abstract]) OR children [Title/Abstract]) OR adolescent [MeSH Terms]) OR child [MeSH Terms]) OR child preschool [MeSH Terms]) OR infant [MeSH Terms])

#3. ((((((prevalence* [Title/Abstract]) OR trend* [Title/Abstract]) OR pattern* [Title/Abstract]) OR monitor* [Title/Abstract]) OR pharmacoepidemiology [Title/Abstract])

#4. ((((((((((medication* [Title/Abstract]) OR medicine* [Title/Abstract]) OR drug* [Title/Abstract]) OR Over-the-Counter [Title/Abstract]) OR Over the Counter [Title/Abstract]) OR claims [Title/Abstract]) OR drug prescription [MeSH Terms]) OR prescription drugs [MeSH Terms]) OR Nonprescription Drugs [MeSH Terms]) OR drug utilization [MeSH Terms]) OR therapeutic use [MeSH Subheading]

#5. ((medication* [Title]) OR drug* [Title]) OR medicine* [Title]

#6. (((((((((hiv [Title/Abstract]) OR hepatitis [Title/Abstract]) OR alcohol [Title/Abstract]) OR illicit [Title/Abstract]) OR inject* [Title/Abstract]) OR cannabis [Title/Abstract]) OR dealer [Title/Abstract]) OR drink* [Title/Abstract]) OR overdos* [Title/Abstract])

#7. ((((#1) AND #2) AND #3) AND #4) AND #5 NOT #6 with filter: publication date: From 2000/01/01

Embase

#1. ('outpatient care'/exp OR 'population health'/exp OR 'pharmacy (shop)'/exp OR 'survey'/exp OR 'outpatient':ti,ab OR 'population-based':ti,ab OR 'noninstitutionalized':ti,ab OR 'nation*':ti,ab OR 'representative':ti,ab OR 'pharmac*':ti,ab OR 'survey':ti,ab OR 'claim*':ti,ab)

#2. ('pediatrics'/exp OR 'pediatrics' OR 'adolescent'/exp OR 'adolescent' OR 'school child'/exp OR 'school child' OR 'preschool child'/exp OR 'preschool child' OR 'infant'/exp OR 'infant' OR 'adolescent*':ti,ab OR 'infan*':ti,ab OR 'children':ti,ab OR 'paediatric':ti,ab OR 'pediatric':ti,ab)

#3. ('prevalence'/exp OR 'drug monitoring'/exp OR 'population statistics'/exp OR 'pharmacoepidemiology'/exp OR 'prevalence':ti,ab OR 'monitor*':ti,ab OR 'pattern*':ti,ab OR 'trend*':ti,ab OR 'pharmacoepidemiology':ti,ab)

#4. ('drug'/exp OR 'drug utilization'/exp OR 'drug use'/exp OR 'administrative claims (health care)'/exp OR 'self medication'/exp OR 'drug*':ti,ab OR 'medication*':ti,ab OR 'medicine*':ti,ab OR 'claim*':ti,ab)

#5. ('drug*':ti OR 'medicine*':ti OR 'medication*':ti)

#6. NOT 'hiv':ti,ab NOT 'hepatitis':ti,ab NOT 'alcohol':ti,ab NOT 'illicit':ti,ab NOT 'inject*':ti,ab NOT 'cannabis':ti,ab NOT 'dealer':ti,ab NOT 'drink*':ti,ab NOT 'overdos*':ti,ab

#7. #1 AND #2 AND #3 AND #4 AND #5 AND #6 AND [humans]/lim AND ([embase]/lim OR [medline]/lim) AND [2000-2020]/py AND ([newborn]/lim OR [infant]/lim OR [child]/lim OR [preschool]/lim OR [school]/lim OR [adolescent]/lim OR [young adult]/lim)

© 2022 Taine M et al. *JAMA Network Open.*

eTable 2. Description of Criteria Used for Extracting Annual Prevalence

Institute (Country)	Website link	Drug tab	Region tab	Sex tab	Age group tab (years)	Sector tab	Indicator tab	ATC levels tab	Ref
NIPH (Norway)	http://www.norpd.no<br><br>Click on “Create a report”	-	Residence: entire country	Sex: Both sexes	Age: 0-4,5-9, 10-14, 15-19	-	Unit of measurement: Users per 1,000 inhabitants	5 levels	[1]
Social-Styrelsen (Sweden)	https://sdb.socialstyrelsen.se/if_lak/val.aspx.	Läkemedelsgrupper och ATC koder för samtliga aldrar<br><br>(Drug classes and ATC codes for all ages)	Regioner: Riket<br><br>(Region: the kingdom)	Kön: Bada könen<br><br>(Sex: both sexes)	Älder (Age): 0-4,5-9, 10-14, 15-19	-	Matt: Patienter/1000 invanare (Patient/1,000 inhabitants)	5 levels	[2]
Zorginstituut Nedderland (The Netherlands)	https://www.gipdatabank.nl/	Categorie : Geneesmiddelen<br><br>(Category: Drugs)	Entire country only [a]	Table of rapportage: Leeftidj en geslacht (0-4, 5-14) (Reporting table: age and sex)		-	-Gegevenssoort: Gebruikers* (Datatype: users*) -Specificatie: Geen specificatie (Specification: no specification)	5 levels	[3]
Sundhedsdata Styrelsen (Denmark)	https://medstat.dk/en	-	Region: Hele landet (Region: entire country)	Kon: Kon, samlet (Sex: overall)	Aldersgruppe (Age group) 0-17	Primaersektor (outpatient)	Sogevariabel : Antal personer pr 1,000 indbyggere (Search variable: Number of persons per 1,000 inhabitants)	5 levels	[4]
AIFA (Italy)	https://www.aifa.gov.it/documents/20142/241052/OsMed_2018_eng.pdf	-	Entire country only [a]	No sex distinction	0-17 years old	-	Users per 100 inhabitants**	Levels 1 and 5	[5]

© 2022 Taine M et al. *JAMA Network Open.*

AIFA, Agenzia Italiana del Farmaco; NIPH, the Norwegian Institute of Public Health

For each website, we completed the platform categories according to the criteria described in the Table above for the year 2019 (except for Italy, which had a report on the AIFA website). We extracted POP prevalences (users per 1000 inhabitants) by age groups and assigned each prevalence a weight according to the proportion of people in that age group in the pediatric population to calculate overall POP prevalence. *For the Dutch study, only the POP user number was available (and not users per 1000 inhabitants). Thus, we searched for the census data in 2019 with same age groups and used them as denominators to calculate the prevalence by age groups and for entire Dutch pediatric population (https://opendata.cbs.nl/statline/#/CBS/en/dataset/37325eng/table?ts=1561370539191).**For Italy, we extracted from the Italian report the pediatric outpatient dispensation prevalences for ATC level 1 drugs and all ATC level 5 drugs.

References

1. Furu K. Establishment of the nationwide Norwegian Prescription Database (NorPD) – new opportunities for research in pharmacoepidemiology in Norway. Vol. 18 No. 2 (2008): Pharmacoepidemiology. https://www.ntnu.no/ojs/index.php/norepid/article/view/23.
2. Socialtyrelsen. Läkemedel - statistik för ar 2014. https://www.socialstyrelsen.se/globalassets/sharepoint-dokument/artikelkatalog/statistik/2015-3-17.pdf.
3. Zorginstituut Nederland. De GIPdatabank 2019. https://www.gipdatabank.nl/veelgestelde-vragen.
4. Pottegard A, Schmidt SAJ, Wallach-Kildemoes H, Sorensen HT, Hallas J, Schmidt M. Data Resource Profile: The Danish National Prescription Registry. *Int J Epidemiol*. Jun 1 2017;46(3):798-798f. doi:10.1093/ije/dyw213
5. Agenzia Italiana del Farmaco. L'uso dei Farmaci in Italia - Rapporto OsMed 2018. https://www.aifa.gov.it/web/guest/-/rapporto-osmed-20-18. Access January 1, 2021.

© 2022 Taine M et al. *JAMA Network Open.*

eTable 3. Anatomical Therapeutic Chemical Level 2 Classes With and Without Nonprescription Drugs

ATC 2	Label	Denmark	France	Italy	The Netherlands	New Zealand	Norway	Sweden
A01	Stomatological preparations	X	X	X	X	X		X
A02	Drugs for acid related disorders	X	X	X	X	X	X	X
A03	Drugs for FGD	X	X	X	X	X	X	X
A04	Antiemetics/antinauseants		X [a]					
A05	Bile and liver therapy							
A06	Drugs for constipation	X	X	X	X	X	X	X
A07	Antidiarrheals	X	X	X	X	X	X	X
A08	Antiobesity preparations.		X	X		X		
A09	Digestives including enzymes							
A10	Drugs used in diabetes							
A11	Vitamins		X	X			X	X
A12	Mineral supplements	X	X	X	X		X	X
B01	Antithrombotic agents		X[c]					
B02	Antihemorrhagics		X	X				X
B03	Antianemic preparations		X	X	X		X	
B05	Blood substitutes		X	X				
C01	Cardiac therapy					X[d]		
C02	Antihypertensives							
C03	Diuretics							
C05	Vasoprotectives	X	X	X	X		X	X
C07	Beta blocking agents							
C08	Calcium channel blockers							
C09	Agents acting on the renin-angiotensin system							
C10	Lipid modifying agents							
D01	Antifungals (topic)	X	X	X	X	X	X	X
D02	Emollients and protectives	X	X	X	X	X	X	X
D04	Antipruritics.	X	X	X		X	X	
D05	Antipsoriatics	X	X			X		X
D06	Antibiotics and chemotherapeutics (topic)	X	X	X	X		X	X
D07	Corticosteroids (topic)		X	X	X	X	X	X
D08	Antiseptics and disinfectants	X	X	X	X		X	X
D09	Medicated dressings	X	X					X
D10	Anti-acne preparations	X	X	X	X	X		X
D11	Other dermatological preparations	X	X	X	X			X
G01	Gynecological antiinfectives and antiseptics	X	X	X		X	X	
G02	Other gynecologicals		X	X				X
G03	Sex hormones	X[e]	X[e]		X[e]	X[e]	X[e]	X[e]
G04	Urologicals		X[f,g]	X[f]			X[g]	X[h]
H01	Pituitary/hypothalamic hormones and analogues							
H02	Corticosteroids (systemic)							
H03	Thyroid therapy							
H04	Pancreatic hormones							
J01	Antibacterials (systemic)							
J02	Antimycotics (systemic)					X[i]	X[i]	
J04	Antimycobacterials							
J05	Antivirals (systemic)			X[j]		X[k]		

© 2022 Taine M et al. *JAMA Network Open.*

ATC 2	Label	Denmark	France	Italy	Netherlands	New Zealand	Norway	Sweden
M01	Antiinflammatory and antirheumatic products	X	X	X	X	X	X	X
M02	Topical products for joint and muscular pain	X	X	X	X		X	X
M03	Muscle relaxants							
N01	Anesthetics	X	X	X			X	
N02	Analgesics	X	X	X	X	X	X	X
N03	Antiepileptics							
N05	Psycholeptics		X[l]	X[l]	X[l]	X[m]	X[l]	X[l]
N06	Psycho-analeptics							
N07	Other nervous system drugs	X	X	X	X		X	X
P01	Antiprotozoals							
P02	Anthelmintics		X		X		X	X
P03	Ectoparasiticides	X	X	X	X			
R01	Nasal preparations	X	X	X	X		X	X
R02	Throat preparations	X	X	X	X		X	X
R03	Drugs for obstructive airway diseases							
R05	Cough and cold preparations	X	X	X	X	X	X	X
R06	Antihistamines for systemic use	X	X	X	X	X	X	X
R07	Other respiratory system products							
S01	Ophthalmologicals	X	X	X	X	X	X	X
S02	Otologicals		X	X	X			
S03	Ophthalmological and otological preparations					X[o]		

ATC, Anatomical Therapeutic Chemical classification

[a] metopimazine; [b] scopolamine; [c] acetylsalicylic acid; [d] epinephrine; [e] emergency contraception; [f] flavoxate; [g] other drugs used in benign prostatic hypertrophy; [h] sildenafil; [i] fluconazole; [j] acyclovir; [k] oseltamivir; [l] Valerianae radix; [m] prochlorperazine; [o] chloramphenicol

Conditions of prescription and reimbursement regarding drugs also available as non-prescription drugs (NPDs) differ greatly between countries (based on age, diseases). For instance, prescribed NPDs such as paracetamol is reimbursed in France regardless of age, reimbursed in Sweden and Norway for chronic disease or specific indications and not reimbursed in Germany after age 12 years except in co-prescription with an opioid. Thus, the international comparisons involving therapeutic classes that include drugs available as NPDs should be interpreted with caution.

We extracted the lists of NPDs in the different countries (see below). A cross indicates that at least one NPD belongs to the therapeutic class in the country.
We classed level 1 and 2 drugs of the ATC classification into two categories:

- The first category consisted of prescription-only drugs such as bile and liver therapy, drugs used in diabetes, antihypertensive agents, diuretics, beta-blocking agents, calcium channel blockers, agents acting on the renin-angiotensin system, pituitary/hypothalamic hormones and analogues, systemic corticosteroids, thyroid therapy, pancreatic hormones, and systemic antibacterial, antimycobacterial, muscle relaxant, antiepileptic, psycho-analeptic, and antiprotozoal agents. We retained cardiac therapy in this category because the only country to have epinephrine available as an NPD was New Zealand and the data for cardiac therapy in New Zealand were not available. Also, some NPDs were not indicated for the pediatric population, such as sildenafil or flaxovate or other drugs used in benign prostatic hypertrophy. Thus, we retained urological agents in this category of prescription-only drugs. Lastly, we considered that emergency contraception, which was an NPD in every country of the study, did not hamper the between-country comparison for the therapeutic class sex hormones, especially for non-emergency contraception.
- The second category did not allow for disentangling prescription-only drug from drugs also available as NPD. The prescription or the non-prescription of drugs also available as NPDs may hamper the comparability and the interpretation of the results.

The lists of NPD were collected on the following websites:
Denmark (2020): https://laegemiddelstyrelsen.dk/en/pharmacies/over-the-counter-medicines/

United Kingdom (2018): https://www.prescqipp.info/umbraco/surface/authorisedmediasurface/index?url=%2fmedia%2f3923%2f227-over-the-counter-items-21.pdf
Italy (2013): http://www.agenziafarmaco.gov.it/content/medicinali-sop-e-otc
The Netherlands (2019): file:///Users/admin/Downloads/MEB21-190510EN-Verkorte+indicaties+op+verpakking+OTC-2.pdf
New Zealand (2010): https://nzpharmacy.wordpress.com/2010/10/01/up-to-date-list-of-pharmacist-only-medicine/
Norway (2020): https://legemiddelverket.no/godkjenning/godkjenning-av-legemidler/reseptfrihet-otc/virkestoffrapporter
Sweden: https://www.lakemedelsverket.se/en/treatment-and-prescription/buy-use-and-handle-medicines/what-is-a-pharmaceutical-product/certain-medicinal-products-for-external-use
https://www.lakemedelsverket.se/sv/handel-med-lakemedel/receptfri-handel-utanfor-apotek/receptfria-lakemedel-som-far-saljas
France: Internal list

© 2022 Taine M et al. *JAMA Network Open.*

eTable 4. Risk of Bias Items Assessed

Risk of bias items	Criteria for answers* and examples	
	**LOW RISK**	**HIGH RISK**
**Internal validity**		
1. Were data collected from a reliable source of prescriptions?	Source of information were prescriptions<br>Example:<br>Source of information was a prescription register	Data were collected from a proxy<br>Example:<br>Source of information was a dispensation claim database
2. Has the exhaustiveness of the study population been taken into account in the building of the prevalence indicator?<br><br>Were users without prescription during the studied period incorporated in the denominator?	Yes<br>Example:<br>-To calculate pediatric national prevalence in 2015, denominator was defined by the census data in 2015 of pediatric population	No<br>Examples:<br>-Denominator was defined by the number of persons having at least 1 prescription in the year.<br>-The denominator of the prevalence was the number of persons having been prescribed ≥1 medication in the previous 5 years
**Threat to generalizability**		
3. Has the sampling frame been restricted on any characteristic?	No<br>Example:<br>The sampling frame was a list of almost every individual within the target population in Lombardy	Yes<br>Example:<br>The sampling frame was a list of just one particular group (only girls, or only residents of a restricted area) within the overall pediatric population
4. Was some form of random selection used to select the sample, or, was a census undertaken to warrant the representativeness of the population in the geographical area?	Yes<br>- Census<br>- Random selection<br>Examples:<br>-The sample was selected using simple random sampling<br>-The target population was a region and every person in the region was sampled	No<br>Example:<br>The study population was children under age 18 enrolled in a nation-wide employer-provided health insurance plan.

* low risk/high risk/unclear

© 2022 Taine M et al. *JAMA Network Open.*

eTable 5. Prevalence of Drug Dispensation and Prescription by Anatomical Therapeutic Chemical Level 2[a]

ATC level 2 label	Code	Sturkenboom et al [19]	Zhang et al [36]	Zhong et al [37]	Zorginstituut Nedderland[40]	Tomlin et al[20]	Taine et al[22]	Sundhedsdata Styrelsen [42]	NIPH [43]	Social-Styrelsen[41]	PR (95% CI)[f]	PD (95% CI)[f]
		3 European countries [b.c]	British Columbia[c]	Olmsted County. MN[c]	The Netherlands[d]	New Zealand	France	Denmark	Norway[e]	Sweden[e]		
		2005	2007	2009	2019	2015	2018-19	2019	2019	2019		
Stomatological preparation	A01	11.2	21.0	-	0.7	20.0	68.4	6.2	2.9	8.0	23.7 (23.7; 23.7)	65.5 (65.4; 65.7)
Drugs for acid disorders	A02	10.1	-	-	6.3	7.4	87.9	9.7	15.9	13.7	9.0 (9.0; 9.1)	78.2 (77.9; 78.4)
Drugs for FGD	A03	12.4	-	10.2 (9.2; 11.2)	3.9	-	149.0	1.1	2.5	2.0	140.6 (140.5; 140.6)	147.9 (147.7; 148.1)
Antiemetic/ antinauseant	A04	2.0	-	-	6.2	34.0	112.9	0.7	1.2	1.3	154.7 (154.6; 154.7)	112.2 (112; 112.3)
Drugs for constipation	A06	15.6	-	-	32.1	32.8	45.4	15.7	20.8	48.2	3.1 (3.1; 3.1)	32.5 (32.2; 32.9)
Antidiarrheals	A07	14.5	-	17.5 (16.2; 18.8)	1.6	-	132.6	5.5	8.6	6.6	24.0 (24.0; 24.0)	127.1(126.9; 127.3)
Vitamins	A11	5.8	-	-	1.7	14.2	303.6	0.2	12.9	8.6	1518 (1518; 1518)	303.4 (303.2; 303.6)
Mineral supplements	A12	1.0	-	-	0.7	14.5	2.3	0.2	0.9	1.7	90.6 (90.5; 90.8)	14.3 (14.1; 14.5)
Antithrombotic agents	B01	0.5	-	-	0.6	-	3.3	0.4	1.2	1.1	8.9 (8.8; 9.0)	2.9 (2.9; 3.0)
Antihemorragics	B02	1.8	-	-	0.4	-	16.5	0.4	0.8	1.8	47.1 (47; 47.2)	16.2 (16.1; 16.2)
Antianemic preparations	B03	7.4	-	-	4.1	-	19.9	1.5	8.5	9.3	13.6 (13.6; 13.7)	18.4 (18.3; 18.5)
Blood substitutes	B05	-	-	-	1.1	-	15.2	0.0	0.6	0.3	506.7 (506.3; 507)	15.2 (15.1; 15.2)
Vasoprotectives	C05		-	-	0.3	-	0.1	2.6	1.1	1.3	25.8 (25.7; 25.9)	2.5 (2.4; 2.6)
Antifungals (topic)	D01	23.0	-	11.3 (10.2; 12.4)	32.4	-	67.7	37.8	17.4	18.4	3.9 (3.9; 3.9)	50.3 (50; 50.5)
Emollients and protectives	D02	63.6	-	-	57.3	113.4	44.1	0.2	58.5	70.4	515.5 (515.3; 515.6)	113.2 (112.7; 113.7)
Antipruritics	D04		-	-	0.0	-	0.0	0.2	1.1	0.5	NA	1.1 (1; 1.1)
Antipsoriatics	D05	4.7	-	-	1.0	26.3	0.8	1.1	0.9	0.6	43.3 (43.2; 43.3)	25.7 (25.4; 26)
Antibiotics+ chemotherapeutics	D06	33.7	33.0	-	75.8	93.7	64.0	33.2	12.5	6.7	14 (13.9; 14)	87.0 (86.5; 87.5)
Corticosteroids	D07	86.5	77.0	76.6 (73.9; 79.3)	81.4	178.9	98.5	79.2	71.4	57.1	3.1 (3.1; 3.1)	121.8 (121.1; 122.5)
Antiseptics and disinfectants	D08	2.8	-	-	0.0	-	155.1	0.0	4.1	1.9	7755 (7755; 7755)	155.1 (154.9; 155.3)
Anti-acne preparations	D10	16.3	33.0	41.4 (39.4; 43.4)	4.0	14.4	33.7	21.0	23.9	16.7	2.3 (2.3; 2.4)	19.3 (19.1; 19.5)
Other dermatologicals preparations	D11	9.1	-	-	3.0	-	0.5	5.4	4.2	2.7	10.7 (10.7; 10.8)	4.9 (4.7; 5.0)

Light and dark grays indicate the lowest and the highest prevalences of level 2 of the ATC classification, respectively. In the last 2 columns, dark gray indicates an ATC level 2 drug with a prevalence ratio (PR) > 2 and a prevalence difference (PD) ≥ 20 pediatric patients per 1000 per year.

NIPH, Norwegian Institute of Public Health; - indicates not available; NA: not applicable

[a] 95% CIs of prevalence numbers were not reported (given the large sample sizes of different studies), except for the study in Olmsted County, Minnesota, USA.

[b] Aggregated data from 3 European countries: Italy (19%), The Netherlands (15%), United Kingdom (66%)

[c] Data are displayed for information purposes, but are not included in the comparison because of their age (ie, ≤2009 or older).

[d] Data are displayed for information purposes, but are not included because of the younger age of the Dutch pediatric population (ie, ages <15 years).

[e] Norway and Sweden include a pediatric population aged less than <20 years.

[f] PR and PD are given between the countries with the highest and lowest prevalence of level 2 of the ATC classification.

© 2022 Taine M et al. *JAMA Network Open.*

eTable 5 (Continued). Prevalence of Drug Dispensation and Prescription by Anatomical Therapeutic Chemical Level 2[a]

ATC level 2 label	Code	Sturkenboom et al [19]	Zhang et al [36]	Zhong et al [37]	Zorginstituut Nedderland[40]	Tomlin et al[20]	Taine et al[22]	Sundhedsdata Styrelsen [42]	NIPH [43]	Social-Styrelsen[41]	PR (95% CI)[f]	PD (95% CI)[f]
		3 European countries [b.c]	British Columbia[c]	Olmsted County. MN[c]	The Netherlands[d]	New Zealand	France	Denmark	Norway[e]	Sweden[e]		
		2005	2007	2009	2019	2015	2018-19	2019	2019	2019		
Gynecological antiinfectives+antiseptics	G01	4.2	-	-	0.9	-	3.6	0.9	2.2	1.4	3.8 (3.8; 3.9)	2.7 (2.6; 2.7)
Other gynecologicals	G02		-	-	0.0	-	0.1	1.1	3.2	4.5	44.8 (44.7; 44.8)	4.4 (4.3; 4.5)
Antimycotics (systemic)	J02	1.8	-	-	1.2	-	8.8	2.1	1.8	2.2	4.9 (4.8; 4.9)	7.0 (6.9; 7.1)
Antivirals systemic use	J05	4.0	-	38.8 (36.9; 40.7)	0.7	-	9.8	5.1	2.3	2.8	4.2 (4.2; 4.2)	7.5 (7.4; 7.6)
Antiinflammatory and antirheumatic products	M01	41.8	-	25.6 (24; 27.2)	15.6	209.5	244.5	20.7	32.7	18.6	13.1 (13.1; 13.1)	225.9 (225.6; 226.2)
Topical products for joint and muscular pain	M02		-	-	0.0	-	31.1	0.2	3.2	0.4	148.1 (148.0; 148.2)	30.9 (30.8; 31)
Anesthetics	N01	4.1	-	-	9.1	-	113.4	0.9	3.1	2.9	120.6 (120.6; 120.7)	112.5 (112.3; 112.6)
Analgesics	N02	63.9	-	-	0.3	434.5	646.7	17.5	25.9	18.9	37.0 (37.0; 37.0)	629.2 (628.9; 629.6)
Psycholeptics	N05	4.5 [b]	-	-	7.4	-	18.7	14.2	24.7	35.3	2.5 (2.5; 2.5)	21.2 (20.8; 21.5)
Other nervous system drugs	N07	1.0	-	-	0.1	-	1.2	0.2	0.1	0.9	9.4 (9.3; 9.6)	1.1 (1.0; 1.1)
Anthelmintics	P02	8.5	-	-	0.1	-	40.6	48.6	1.7	30.4	28.2 (28.1; 28.2)	46.9 (46.5; 47.3)
Ectoparasiticides	P03	13.4	-	-	1.9	-	5.8	5.2	1.1	1.1	5.4 (5.4; 5.4)	4.7 (4.7; 4.8)
Nasal preparations	R01	46.1	25.0	-	7.7	40.6	331.4	39.2	64.0	36.9	9.0 (9.0; 9.0)	294.5 (294.1; 294.9)
Throat preparations	R02	2.3	-	-	0.0	-	0.6	0.4	0.1	0.1	6.2 (6.1; 6.4)	0.5 (0.5; 0.5)
Cough & cold preparations	R05	2.3	-	11.8 (10.7; 12.9)	1.4	-	173.4	1.8	30.5	60.4	96.3 (96.3; 96.4)	171.6 (171.4; 171.8)
Antihistamines	R06	29.7	-	52.2 (50.0; 54.4)	60.0	178.4	245.9	41.2	114.2	90.0	6.0 (6.0; 6.0)	204.7 (204.3; 205.2)
Ophtalmologicals	S01	69.0	94.0	-	51.6	96.9	140.1	97.5	102.1	44.3	3.2 (3.2; 3.2)	95.8 (95.5; 96.1)
Otologicals	S02	15.1	-	-	26.6	-	98.3	34.9	5.1	1.9	51.8 (51.7; 51.8)	96.4 (96.2; 96.6)
Ophtalmological and otological preparations	S03	3.8	-	-	-	14.8	-	5.9	13.6	23.2	3.9 (3.9; 4.0)	17.3 (17.1; 17.5)

Light and dark grays indicate the lowest and the highest prevalence of level 2 of the ATC classification, respectively. In the last 2 columns, dark gray indicates an ATC level 2 drug with a prevalence ratio (PR) > 2 and a prevalence difference (PD) ≥ 20 pediatric patients per 1000 per year.

NIPH, Norwegian Institute of Public Health; - indicates not available; NA: not applicable

[a] 95% CIs of prevalence numbers were not reported (given the large sample sizes of different studies), except for the study in Olmsted County, Minnesota, USA.

[b] Aggregated data from 3 European countries: Italy (19%), The Netherlands (15%), United Kingdom (66%)

[c] Data are displayed for information purposes, but are not included in the comparison because of their age (ie, ≤2009 or older).

[d] Data are displayed for information purposes, but are not included because of the younger age of the Dutch pediatric population (ie, ages <15 years).

[e] Norway and Sweden include a pediatric population aged less than <20 years.

[f] PR and PD are given between the countries with the highest and lowest prevalence of level 2 of the ATC classification.

© 2022 Taine M et al. *JAMA Network Open.*

eTable 6. Prevalence of Drug Dispensation and Prescription by Anatomical Therapeutic Chemical Level 1[a]

Code	ATC level 1 drugs	Sturkenboom et al [19]	Zorginstituut Nedderland[40]	Tomlin et al[20]	AIFA [39]	Taine et al[22]	Sundhedsdata Styrelsen [42]	NIPH [43]	Social-Styrelsen[41]	PR (95% CI)[e]	PD (95% CI)[e]
		3 European countries[b,c]	The Netherlands[c]	New Zealand[d]	Italy	France	Denmark	Norway	Sweden		
		2005	2019	2010-2015	2018	2018-19	2019	2019	2019		
A	Alimentary tract and metabolism*	58	62	90	53	516	39	62	83	13.2 (13.2; 13.2)	477.0 (476.6; 477.4)
B	Blood/blood forming organs*	12	6	53	-	52	2	11	12	26.5 (26.5; 26.5)	51 (50.8; 51.2)
C	Cardiovascular*	8	3	3	-	7	6	13	12	4.3 (4.3; 4.4)	10.0 (9.8; 10.2)
D	Dermatologicals*	172	51	324	-	318	153	143	124	2.6 (2.6; 2.6)	200 (199.4; 200.6)
G	Genito urinary system and sex hormones*	37	10[b]	35	-	24	45	61	52	2.5 (2.5; 2.5)	37 (36.6; 37.4)
H	Systemic hormonal preparations	26	10	84	88	216	9	22	25	24.0 (24.0; 24.0)	207.0 (206.7; 207.3)
J	Anti-infective (systemic)*	283	73	495	387	412	189	156	147	3.4 (3.4; 3.4)	348 (347.4; 348.6)
L	Antineoplastic and immunomodulating agents	5	1	Low	-	2	1	3	3	3.0 (2.9; 3.1)	2.0 (1.9; 2.1)
M	Musculo-skeletal system*	46	16	180	-	256	21	35	20	12.2 (12.2; 12.2)	235 (234.7; 235.3)
N	Nervous system*	69	30	458	9	672	45	65	72	74.7 (74.7; 74.7)	663 (662.8; 663.2)
P	Antiparasitic products*	25	4	-	-	46	59	8	36	7.4 (7.4; 7.4)	51 (50.5; 51.5)
R	Respiratory system*	198	47	269	198	499	121	197	194	4.1 (4.1; 4.1)	378 (377.4; 378.6)
S	Sensory organs*	82	57	118	-	215	129	117	66	3.3 (3.3; 3.3)	149.0 (148.8; 149.2)

AIFA, Agenzia Italiana del Farmaco; NIPH, Norwegian Institute of Public Health

[a] 95% confidence intervals (CI) of prevalences were not reported given the large sample sizes of different studies

[b] Aggregated data from 3 European countries: Italy (19%), The Netherlands (15%), United Kingdom (66%)

[c] Data are displayed for information purposes, but are not included in the comparison because of their age (ie, ≤2009 or older) or of the younger age of the Dutch pediatric population (ie, ages <15 years).

[d] Comparisons with New Zealand data should be interpreted with caution because they correspond to mean prevalences from 2010 to 2015 (and not 2015 prevalence only as for ATC level 2 and 5 drugs)

[e] PR and PD are given between the countries with the highest and lowest prevalence of level 1 of the ATC classification. Light and dark grays indicate the lowest and the highest prevalence of level 1 of the ATC classification, respectively.

[*] These comparisons should be interpreted with caution because level 1 of the ATC classification includes non-prescription drugs for which the conditions of prescription and reimbursement differ greatly among countries (based on age, diseases, or without reimbursement)

Low indicates below <3 pediatric patients per 1000 per year; - indicates not available

© 2022 Taine M et al. *JAMA Network Open.*

eTable 7. Prevalence of Drug Dispensation and Prescription by Anatomical Therapeutic Chemical Level 2 in Same Age Group Studies

ATC level 2 label	Code	<18 years old					<20 years old			
		Tomlin et al[20]	Taine et al[22]	Sundhedsdata Styrelsen [42]			NIPH[43]	Social-Styrelsen[41]		
		New Zealand	France	Denmark	PR (95% CI)[a]	PD (95% CI)[a]	Norway	Sweden	PR (95% CI)[a]	PD (95% CI)[a]
		2015	2018-19	2019			2019	2019		
Bile and liver therapy	A05	-	0.5	0.1	4.8 (4.8; 4.9)	0.4 (0.4; 0.5)	0.1	0.2	1.6 (1.4; 1.7)	0.1 (0.1; 0.1)
Digestives (including enzymes)	A09	-	0.2	0.2	1.1 (1; 1.2)	0 (0; 0)	0.1	0.2	1.6 (1.4; 1.8)	0.1 (0.1; 0.1)
Drugs used in diabetes	A10	-	1.8	2.7	1.5 (1.5; 1.5)	0.9 (0.8; 1)	3.3	4	1.2 (1.2; 1.2)	0.7 (0.6; 0.8)
Cardiac therapy	C01	-	5.1	1.7	3 (3; 3)	3.4 (3.4; 3.5)	8.3	5.6	1.5 (1.5; 1.5)	2.7 (2.6; 2.9)
Antihypertensives	C02	-	0.1	0.3	3.2 (3.1; 3.3)	0.2 (0.2; 0.2)	0.5	2.9	5.7 (5.7; 5.8)	2.4 (2.3; 2.5)
Diuretics	C03	-	0.3	0.3	1.3 (1.2; 1.3)	0.1 (0; 0.1)	0.3	0.4	1.3 (1.1; 1.4)	0.1 (0; 0.1)
Beta blocking agents	C07	-	1.2	1.1	1.2 (1.1; 1.2)	0.2 (0.1; 0.2)	1.5	1.7	1.1 (1.0; 1.1)	0.1 (0; 0.2)
Calcium channel blockers	C08	-	0.2	0.2	1.5 (1.4; 1.6)	0.1 (0.1; 0.1)	0.3	0.2	1.3 (1.2; 1.5)	0.1 (0; 0.1)
Agents acting on the RA system	C09	-	0.5	0.5	1.1 (1; 1.1)	0 (0; 0.1)	1.2	0.7	1.7 (1.6; 1.7)	0.5 (0.4; 0.5)
Lipid modifying agents	C10	-	0.2	0.2	1.4 (1.3; 1.5)	0.1 (0; 0.1)	0.7	0.2	2.9 (2.8; 3)	0.4 (0.4; 0.5)
Sex hormones	G03	22	20.6	42.5	2.1 (2.1; 2.1)	21.9 (21.7; 22.1)	57.2	47.3	1.2 (1.2; 1.2)	9.9 (9.5; 10.4)
Urologicals	G04	-	1.7	1.4	1.2 (1.1; 1.2)	0.2 (0.2; 0.3)	1.4	1.8	1.3 (1.2; 1.3)	0.4 (0.3; 0.5)
Pituitary/ hypothalamic hormones	H01	-	4.2	5.2	1.2 (1.2; 1.2)	1 (0.9; 1)	8.7	5.1	1.7 (1.7; 1.7)	3.6 (3.4; 3.8)
Corticosteroids for systemic use	H02	82	209.9	1.9	108.2 (108.2; 108.2)	208 (207.5; 208.5)	9.9	15.9	1.6 (1.6; 1.6)	6.0 (5.7; 6.2)
Thyroid therapy	H03	-	1.7	1.3	1.3 (1.2; 1.3)	0.4 (0.3; 0.4)	2.5	3.2	1.3 (1.2; 1.3)	0.6 (0.5; 0.7)
Pancreatic hormones	H04	-	1.5	1	1.6 (1.6; 1.6)	0.6 (0.5; 0.6)	1.7	1.4	1.2 (1.1; 1.2)	0.2 (0.2; 0.3)
Antibacterials for systemic use	J01	480	404.8	171.3	2.8 (2.8; 2.8)	308.7 (307.7; 309.6)	142.7	141.2	1.0 (1.0; 1.0)	1.5 (0.8; 2.2)
Antimycobacterials	J04	-	0.5	0.3	18.2 (18; 18.3)	0.5 (0.5; 0.5)	0.2	0.2	1.0 (0.8; 1.1)	0 (0; 0)
Antineoplastic agents	L01	-	0.5	0.2	2.2 (2.1; 2.3)	0.3 (0.2; 0.3)	0.4	0.3	1.5 (1.3; 1.6)	0.1 (0.1; 0.2)
Immunosuppressants	L04	-	0.9	0.5	1.7 (1.7; 1.8)	0.4 (0.4; 0.4)	2.1	2.1	1.0 (1.0; 1.1)	0 (0; 0.1)
Muscle relaxants	M03	-	0.3	0.6	2.3 (2.2; 2.4)	0.3 (0.3; 0.3)	0.3	1.4	4.6 (4.5; 4.7)	1.1 (1.0; 1.2)
Antiepileptics	N03	-	3.9	3.6	1.1 (1.1; 1.1)	0.3 (0.2; 0.3)	5.1	5.2	1.0 (1.0; 1.0)	0.1 (0; 0.2)
Psycho-analeptics	N06	11	7.9	17.9	2.3 (2.3; 2.3)	10 (9.9; 10.1)	21.9	36.2	1.6 (1.6; 1.7)	14.2 (13.9; 14.6)
Antiprotozoals	P01	-	3	5.3	1.7 (1.7; 1.7)	2.2 (2.2; 2.3)	5.4	5	1.1 (1.0; 1.1)	0.3 (0.2; 0.5)
Drugs for obstructive airway diseases	R03	131	143.2	67.3	2.1 (2.1; 2.1)	76 (75.6; 76.4)	70.4	80.6	1.1 (1.1; 1.2)	10.1 (9.6; 10.7)

Light and dark grays indicate the lowest and the highest prevalence of level 2 of the ATC classification, respectively. [a]Dark gray indicates an ATC level 2 drug with a prevalence ratio (PR) > 2 and a prevalence difference (PD) ≥ 20 pediatric patients per 1000 per year.

NIPH, Norwegian Institute of Public Health; - indicates not available; NA: not applicable

© 2022 Taine M et al. *JAMA Network Open.*

eTable 8. Prevalence of Drug Dispensation and Prescription by Anatomical Therapeutic Chemical Level 2 Among Children Aged Less Than 5 to 6 Years[a]

ATC level 2 label	Code	Tomlin et al[20]	Taine et al[22]	Sundhedsdata Styrelsen [42]	Zorginstituut Nedderland[40]	NIPH[43]	Social-Styrelsen[41]	PR (95% CI)[c]	PD (95% CI)[c]
		New Zealand	France	Denmark	The Netherlands[b]	Norway	Sweden		
		2015	2018-19	2019	2019	2019	2019		
Bile and liver therapy	A05	-	0.2	0.2	0.1	0.2	0.3	2.5 (2.3; 2.8)	0.2 (0.1; 0.2)
Digestives (including enzymes)	A09	-	0.2	0.2	0.2	0.1	0.2	2.1 (1.6; 2.6)	0.1 (0; 0.2)
Drugs used in diabetes	A10	-	0.5	0.6	0.2	0.4	0.5	2.7 (2.5; 2.9)	0.4 (0.3; 0.5)
Cardiac therapy	C01	-	3.8	2.3	3.0	6.6	2.6	2.9 (2.8; 3.0)	4.3 (4.0; 4.7)
Antihypertensives	C02		0.0	0.0	0.0	0.0	0.0	NA	0.0 (0.0; 0.1)
Diuretics	C03	-	0.5	0.3	0.6	0.6	0.7	2.4 (2.2; 2.7)	0.4 (0.3; 0.5)
Beta blocking agents	C07	-	0.7	0.5	1.0	1.3	1.1	2.4 (2.2; 2.6)	0.7 (0.6; 0.9)
Calcium channel blockers	C08	-	0.1	0.0	0.2	0.2	0.1	NA	0.2 (0.2; 0.2)
Agents acting on the RA system	C09	-	0.2	0.1	0.2	0.5	0.3	6.8 (6.4; 7.2)	0.5 (0.4; 0.6)
Lipid modifying agents	C10	-	0.0	0.0	0.0	0.0	0.0	NA	0 (0; 0)
Urologicals	G04	-	0.4	0.2	0.7	0.1	0.2	6.4 (6.0; 6.7)	0.6 (0.5; 0.7)
Pituitary/ hypothalamic hormones	H01		0.6	0.6	0.1	0.6	0.2	11.8 (11.7; 11.0)	0.6 (0.5; 0.7)
Corticosteroids for systemic use	H02	136.3	328.7	0.5	6.1	10.0	12.9	655.2 (655.1; 655.4)	328.2 (328.6; 328.6)
Thyroid therapy	H03	-	1.0	0.5	0.7	1.0	0.9	1.9 (1.7; 2)	0.5 (0.4; 0.5)
Pancreatic hormones	H04		0.3	0.2	0.2	0.3	0.3	2.0 (1.9; 2.2)	0.2 (0.1; 0.2)
Antibacterials for systemic use	J01	628.6	565.5	245.1	118.1	190.4	187.0	5.3 (5.3; 5.3)	510.5 (508; 513)
Antimycobacterials	J04	-	0.6	0.0	0.2	0.1	0.1	NA	0.6 (0.6; 0.7)
Antineoplastic agents	L01	-	0.3	0.5	0.1	0.2	0.2	4.3 (4.1; 4.6)	0.4 (0.3; 0.5)
Immunosuppressants	L04	-	0.3	0.1	0.3	0.5	0.5	3.9 (3.5; 4.2)	0.4 (0.3; 0.5)
Muscle relaxants	M03	-	0.0	0.3	0.1	0.1	0.1	8.6(8.3; 8.8)	0.2 (0.2; 0.3)
Antiepileptics	N03	-	2.4	2.1	1.5	2.3	2.1	1.6 (1.5; 1.6)	0.9 (0.8; 1)
Psycho-analeptics	N06	-	0.6	0.3	0.1	0.0	0.1	NA	0.6 (0.5; 0.6)
Antiprotozoals	P01	-	2.1	2.3	1.7	2.4	2.9	1.7 (1.6; 1.7)	1.2 (1.0; 1.3)
Drugs for obstructive airway diseases	R03	-	213.1	108.9	96.0	92.9	106.3	2.3 (2.3; 2.3)	120.2 (119.1; 121.3)

Light and dark grays indicate the lowest and the highest prevalence of level 2 of the ATC classification, respectively. In the last 2 columns, dark gray indicates an ATC level 2 drug with a prevalence ratio (PR) > 2 and a prevalence difference (PD) ≥ 20 pediatric patients per 1000 per year.

NIPH, Norwegian Institute of Public Health; - indicates not available; NA: not applicable

[a] 95% CIs of prevalence numbers were not reported given the large sample sizes of different studies.

[b] The Dutch study is included in the comparative analyses for children<5 to 6 years old because this Dutch age group is similar as the other studies

[c] PR and PD are given between the countries with the highest and lowest prevalence of level 2 of the ATC classification.

© 2022 Taine M et al. *JAMA Network Open.*

eTable 9: Prevalence of Drug Dispensation and Prescription by Anatomical Therapeutic Chemical Level 2 Among Children Aged Less Than 5 to 6 Years in Same Age Group Studies

ATC level 2 label	Code	<6 years old					<5 years old				
		Tomlin et al[20]	Taine et al[22]	Sundhedsdata Styrelsen [42]	PR (95% CI)[b]	PD (95% CI)[b]	Zorginstituut Nedderland[40]	NIPH[43]	Social-Styrelsen[41]	PR (95% CI)[b]	PD (95% CI)[b]
		New Zealand	France	Denmark			The Netherlands[c]	Norway	Sweden		
		2015	2018-19	2019			2019	2019	2019		
Bile and liver therapy	A05	-	0.2	0.2	1.1 (0.9; 1.4)	0 (0; 0.1)	0.1	0.2	0.3	2.5 (2.3; 2.8)	0.2 (0.1; 0.2)
Digestives (including enzymes)	A09	-	0.2	0.2	1 (0.7; 1.2)	0 (0; 0)	0.2	0.1	0.2	1.8 (1.3; 2.2)	0.1 (0.0; 0.1)
Drugs used in diabetes	A10	-	0.5	0.6	1.3 (1.2; 1.4)	0.1 (0.1; 0.2)	0.2	0.4	0.5	2.4 (2.3; 2.6)	0.3 (0.3; 0.4)
Cardiac therapy	C01	-	3.8	2.3	1.7 (1.6; 1.8)	1.6 (1.4; 1.7)	3.0	6.6	2.6	2.5 (2.5; 2.6)	4 (3.7; 4.3)
Antihypertensives	C02		0.0	0.0	NA	0 (0; 0)	0.0	0.0	0.0	2.2 (1.2; 3.1)	0 (0; 0)
Diuretics	C03	-	0.5	0.3	1.5 (1.3; 1.7)	0.2 (0.1; 0.2)	0.6	0.6	0.7	1.2 (1.0; 1.4)	0.1 (0; 0.2)
Beta blocking agents	C07	-	0.7	0.5	1.3 (1.1; 1.4)	0.1 (0.1; 0.2)	1.0	1.3	1.1	1.2 (1.1; 1.4)	0.2 (0.1; 0.4)
Calcium channel blockers	C08	-	0.1	0.0	NA	0.1 (0.1; 0.1)	0.2	0.2	0.1	2.4 (2.1; 2.7)	0.1 (0.1; 0.2)
Agents acting on the RA system	C09	-	0.2	0.1	2.1 (1.8; 2.5)	0.1 (0.1; 0.1)	0.2	0.5	0.3	2.3 (2.1; 2.5)	0.3 (0.2; 0.4)
Lipid modifying agents	C10	-	0.1	0.0	10 (9; 11)	0.1 (0.1; 0.1)	0.0	0.0	0.0	NA	0 (0; 0)
Urologicals	G04	-	0.4	0.2	1.6 (1.4; 1.8)	0.1 (0.1; 0.2)	0.7	0.1	0.2	6.4 (6; 6.7)	0.6 (0.5; 0.7)
Pituitary/ hypothalamic hormones	H01		0.6	0.6	1.1 (1; 1.2)	0.1 (0; 0.1)	0.1	0.6	0.2	10.5 (10.2; 10.9)	0.5 (0.4; 0.6)
Corticosteroids for systemic use	H02	136.3	328.7	0.5	655.2 (655.1; 655.4)	328.2 (327.8;328.6)	6.1	10.0	12.9	2.1 (2.1; 2.2)	6.9 (6.5; 7.2)
Thyroid therapy	H03	-	1.0	0.5	1.9 (1.7; 2)	0.5 (0.4; 0.5)	0.7	1.0	0.9	1.4 (1.3; 1.6)	0.3 (0.1; 0.4)
Pancreatic hormones	H04		0.3	0.2	1.8 (1.6; 2.1)	0.2 (0.1; 0.2)	0.2	0.3	0.3	2 (1.8; 2.3)	0.2 (0.1; 0.2)
Antibacterials for systemic use	J01	628.6	565.5	245.1	2.6 (2.6; 2.6)	383.5 (381.4;385.6)	118.1	190.4	187.0	1.6 (1.6; 1.6)	72.3 (70.7; 74)
Antimycobacterials	J04	-	0.6	0.0	NA	0.6 (0.6; 0.7)	0.2	0.1	0.1	3.3 (2.8; 3.8)	0.1 (0.1; 0.2)
Antineoplastic agents	L01	-	0.3	0.5	1.8 (1.6; 1.9)	0.2 (0.1; 0.3)	0.1	0.2	0.2	2 (1.7; 2.3)	0.1 (0.1; 0.2)
Immunosuppressants	L04	-	0.3	0.1	2.4 (2.1; 2.7)	0.2 (0.2; 0.2)	0.3	0.5	0.5	1.9 (1.7; 2.1)	0.3 (0.2; 0.3)
Muscle relaxants	M03	-	0.0	0.3	8.6 (8.3; 8.8)	0.2 (0.2; 0.3)	0.1	0.1	0.1	1.5 (1.2; 1.8)	0.0 (0.0; 0.1)
Antiepileptics	N03	-	2.4	2.1	1.2 (1.1; 1.2)	0.3 (0.2; 0.5)	1.5	2.3	2.1	1.5 (1.4; 1.6)	0.8 (0.6; 1)
Psycho-analeptics	N06	-	0.6	0.3	2 (1.8; 2.2)	0.3 (0.2; 0.3)	0.1	0.0	0.1	NA	0.1 (0.1; 0.1)
Antiprotozoals	P01	-	2.1	2.3	1.1 (1; 1.1)	0.1 (0; 0.3)	1.7	2.4	2.9	1.7 (1.6; 1.7)	1.2 (1; 1.3)
Drugs for obstructive airway diseases	R03	-	213.1	108.9	2 (1.9; 2)	104.2 (103.1; 105.3)	96.0	92.9	106.3	1.1 (1.1; 1.2)	13.4 (12; 14.7)

Light and dark grays indicate the lowest and the highest prevalence of level 2 of the ATC classification, respectively. NIPH, Norwegian Institute of Public Health;

[a] 95% confidence intervals (CI) of prevalences were not reported given the large sample sizes of different studies

[b] PR and PD are given between the countries with the highest and lowest prevalence of level 2 of the ATC classification. Dark gray indicates an ATC level 2 drug with a prevalence ratio (PR) > 2 and a prevalence difference (PD) ≥ 20 pediatric patients per 1000 per year.

© 2022 Taine M et al. *JAMA Network Open.*

eTable 10. Prevalence of Drug Dispensation and Prescription by Anatomical Therapeutic Chemical Level 2 Among Pediatric Patients Aged 5 to 6 Years or Older[a]

ATC level 2 label	Code	Zorginstituut Nedderland[40]	Tomlin et al[20]	Taine et al[22]	Sundhedsdata Styrelsen [42]	NIPH[43]	Social-Styrelsen[41]	PR (95% CI)[c]	PD (95% CI)[c]
		The Netherlands[b]	New Zealand	France	Denmark	Norway	Sweden		
		2019	2015	2018-19	2019	2019	2019		
Bile and liver therapy	A05	0.1	-	0.7	0.1	0.1	0.2	5.2 (5.0; 5.4)	0.6 (0.5; 0.6)
Digestives (including enzymes)	A09	0.2	-	0.2	0.2	0.1	0.2	1.6 (1.4; 1.8)	0.1 (0.0; 0.1)
Drugs used in diabetes	A10	2.0	-	2.3	3.6	4.1	5.3	2.3 (2.2; 2.3)	2.9 (2.8; 3.1)
Cardiac therapy	C01	4.8	-	5.6	1.5	8.8	6.6	6.1 (6; 6.1)	7.4 (7.2; 7.6)
Antihypertensives	C02	0.5		0.1	0.4	0.6	3.9	35.5 (35.4; 35.5)	3.8 (3.7; 3.9)
Diuretics	C03	0.2	-	0.3	0.2	0.2	0.2	1.5 (1.4; 1.6)	0.1 (0.1; 0.1)
Beta blocking agents	C07	0.8	-	1.5	1.3	1.6	1.9	1.5 (1.5; 1.6)	0.7 (0.6; 0.8)
Calcium channel blockers	C08	0.2	-	0.3	0.2	0.3	0.3	1.3 (1.1; 1.5)	0.1 (0.0; 0.1)
Agents acting on the RA system	C09	0.6	-	0.7	0.6	1.3	0.8	2.1 (2.0; 2.2)	0.7 (0.6; 0.8)
Lipid modifying agents	C10	0.4	-	0.3	0.2	0.8	0.3	4 (3.8; 4.2)	0.6 (0.6; 0.7)
Sex hormones	G03	10.5[b]	-	29.2	60.7	72.9 [b]	66.6 [b]	2.5 (2.5; 2.5)	43.7 (43.3; 44.3)
Urologicals	G04	2.2	-	2.3	2.0	1.7	2.3	1.3 (1.3; 1.4)	0.6 (0.5; 0.7)
Pituitary/ hypothalamic hormones	H01	3.2		5.9	7.3	10.9	6.6	1.9 (1.8; 1.9)	5.0 (4.8; 5.2)
Corticosteroids for systemic use	H02	5.4	49.6	156.1	2.6	9.9	17.0	60.9 (60.8; 60.9)	153.6 (153.3; 153.8)
Thyroid therapy	H03	1.4	-	2.0	1.6	2.9	4.0	2.5 (2.4; 2.5)	2.4 (2.3; 2.5)
Pancreatic hormones	H04	1.1		2.0	1.3	2.0	1.8	1.5 (1.5; 1.6)	0.7 (0.6; 0.8)
Antibacterials for systemic use	J01	55.2	412.5	332.0	137.0	129.9	125.8	3.3 (3.3; 3.3)	286.7 (285.4; 287.9)
Antimycobacterials	J04	0.2	-	0.5	0.0	0.2	0.2	24 (23.5; 24.5)	0.5 (0.4; 0.5)
Antineoplastic agents	L01	0.2	-	0.6	0.1	0.4	0.3	7.3 (7.1; 7.6)	0.5 (0.5; 0.5)
Immunosuppressants	L04	1.0	-	1.2	0.7	2.6	2.7	3.8 (3.8; 3.9)	2.0 (1.9; 2.1)
Muscle relaxants	M03	0.2	-	0.4	0.7	0.4	1.9	5.3 (5.2; 5.4)	1.6 (1.5; 1.6)
Antiepileptics	N03	1.9	-	4.6	4.3	5.9	6.3	1.5 (1.4; 1.5)	2 (1.8; 2.1)
Psycho-analeptics	N06	35.3	-	11.2	25.6	27.9	49.8	4.4 (4.4; 4.4)	38.6 (38.2; 38.9)
Antiprotozoals	P01	2.6	-	3.5	6.7	6.2	5.8	1.9 (1.9; 2.0)	3.2 (3.0; 3.4)
Drugs for obstructive airway diseases	R03	56.4	-	111.5	47.7	64.2	71.6	2.3 (2.3; 2.3)	63.8 (63.3; 64.3)

Light and dark grays indicate the lowest and the highest prevalence of level 2 of the ATC classification, respectively. In the last 2 columns, dark gray indicates an ATC level 2 drug with a prevalence ratio (PR) > 2 and a prevalence difference (PD) ≥ 20 pediatric patients per 1000 per year.

NIPH, Norwegian Institute of Public Health; [a] 95% confidence intervals (CI) of prevalences were not reported given the large sample sizes of different studies; - indicates not available; NA: not applicable

[a] 95% confidence intervals (CI) of prevalences were not reported given the large sample sizes of different studies

[b] Data are displayed for information purposes, but are not included because of the younger age of the Dutch pediatric population (ie, ages <15 years).

[c] PR and PD are given between the countries with the highest and lowest prevalence of level 2 of the ATC classification.

© 2022 Taine M et al. *JAMA Network Open.*

eTable 11. Prevalence of Drug Dispensation and Prescription by Anatomical Therapeutic Chemical Level 2 Among Pediatric Patients Aged 5 to 6 Years or Older in Same Age Group Studies

ATC level 2 label	Code	6-17 years old					5-19 years old			
		Tomlin et al[20] New Zealand 2015	Taine et al[22] France 2018-19	Sundhedsdata Styrelsen [42] Denmark 2019	PR (95% CI)[b]	PD (95% CI)[b]	NIPH[43] Norway 2019	Social-Styrelsen[41] Sweden 2019	PR (95% CI)[b]	PD (95% CI)[b]
Bile and liver therapy	A05	-	0.7	0.1	5.2 (5.0; 5.4)	0.6 (0.5; 0.6)	0.1	0.2	1.6 (1.4; 1.8)	0.1 (0; 0.1)
Digestives (including enzymes)	A09	-	0.2	0.2	1.2 (1.0; 1.3)	0 (0; 0.1)	0.1	0.2	1.6 (1.4; 1.8)	0.1 (0.1; 0.1)
Drugs used in diabetes	A10	-	2.3	3.6	1.6 (1.5; 1.6)	1.3 (1.2; 1.4)	4.1	5.3	1.3 (1.3; 1.3)	1.2 (1; 1.4)
Cardiac therapy	C01	-	5.6	1.5	3.9 (3.8; 3.9)	4.2 (4.1; 4.3)	8.8	6.6	1.3 (1.3; 1.4)	2.2 (2.0; 2.4)
Antihypertensives	C02		0.1	0.4	3.8 (3.7; 3.9)	0.3 (0.3; 0.4)	0.6	3.9	6.2 (6.1; 6.3)	3.3 (3.2; 3.4)
Diuretics	C03	-	0.3	0.2	1.2 (1.1; 1.4)	0.1 (0; 0.1)	0.2	0.2	1.2 (1.0; 1.4)	0 (0; 0.1)
Beta blocking agents	C07	-	1.5	1.3	1.1 (1.1; 1.2)	0.2 (0.1; 0.3)	1.6	1.9	1.2 (1.1; 1.2)	0.3 (0.2; 0.4)
Calcium channel blockers	C08	-	0.3	0.2	1.3 (1.2; 1.5)	0.1 (0; 0.1)	0.3	0.3	1.2 (1.0; 1.3)	0 (0; 0.1)
Agents acting on the RA system	C09	-	0.7	0.6	1.1 (1.0; 1.2)	0 (0; 0.1)	1.3	0.8	1.6 (1.5; 1.7)	0.5 (0.4; 0.6)
Lipid modifying agents	C10	-	0.3	0.2	1.3 (1.2; 1.5)	0.1 (0; 0.1)	0.8	0.3	2.8 (2.7; 2.9)	0.5 (0.5; 0.6)
Sex hormones	G03	-	29.2	60.7	2.1 (2.1; 2.1)	31.5 (30.9; 32)	72.9 [b]	66.6 [b]	1.1 (1.1; 1.1)	6.3 (5.7; 7.0)
Urologicals	G04	-	2.3	2.0	1.1 (1.1; 1.2)	0.3 (0.2; 0.4)	1.7	2.3	1.3 (1.3; 1.4)	0.6 (0.5; 0.7)
Pituitary/ hypothalamic hormones	H01		5.9	7.3	1.2 (1.2; 1.3)	1.4 (1.2; 1.6)	10.9	6.6	1.6 (1.6; 1.7)	4.3 (4.1; 4.5)
Corticosteroids for systemic use	H02	49.6	156.1	2.6	60.9 (60.8; 60.9)	153.6 (153.3; 153.8)	9.9	17.0	1.7 (1.7; 1.7)	7.1 (6.8; 7.3)
Thyroid therapy	H03	-	2.0	1.6	1.2 (1.2; 1.3)	0.4 (0.3; 0.5)	2.9	4.0	1.4 (1.3; 1.4)	1.1 (0.9; 1.2)
Pancreatic hormones	H04		2.0	1.3	1.5 (1.4; 1.6)	0.7 (0.6; 0.7)	2.0	1.8	1.1 (1.1; 1.2)	0.2 (0.1; 0.3)
Antibacterials for systemic use	J01	412.5	332.0	137.0	3.0 (3.0; 3.0)	275.5 (274.1; 276.9)	129.9	125.8	1.0 (1.0; 1.0)	4.1 (3.2; 4.9)
Antimycobacterials	J04	-	0.5	0.0	24 (23.5; 24.5)	0.5 (0.4; 0.5)	0.2	0.2	1.0 (0.9; 1.2)	0 (0; 0)
Antineoplastic agents	L01	-	0.6	0.1	7.3 (7.1; 7.6)	0.5 (0.5; 0.5)	0.4	0.3	1.4 (1.3; 1.6)	0.1 (0.1; 0.2)
Immunosuppressants	L04	-	1.2	0.7	1.6 (1.5; 1.7)	0.5 (0.4; 0.5)	2.6	2.7	1.1 (1.0; 1.1)	0.2 (0; 0.3)
Muscle relaxants	M03	-	0.4	0.7	1.9 (1.8; 1.9)	0.3 (0.3; 0.4)	0.4	1.9	5.3 (5.2; 5.4)	1.6 (1.5; 1.6)
Antiepileptics	N03	-	4.6	4.3	1.1 (1.0; 1.1)	0.2 (0.1; 0.4)	5.9	6.3	1.1 (1.0; 1.1)	0.4 (0.2; 0.6)
Psycho-analeptics	N06	-	11.2	25.6	2.3 (2.3; 2.3)	14.3 (14; 14.7)	27.9	49.8	1.8 (1.8; 1.8)	21.9 (21.5; 22.4)
Antiprotozoals	P01	-	3.5	6.7	1.9 (1.9; 2.0)	3.2 (3.0; 3.4)	6.2	5.8	1.1 (1; 1.1)	0.3 (0.2; 0.5)
Drugs for obstructive airway diseases	R03	-	111.5	47.7	2.3 (2.3; 2.3)	63.8 (63.3; 64.3)	64.2	71.6	1.1 (1.1; 1.1)	7.3 (6.7; 7.9)

Light and dark grays indicate the lowest and the highest prevalence of level 2 of the ATC classification, respectively.

NIPH, Norwegian Institute of Public Health; - indicates not available

[a] 95% confidence intervals (CI) of prevalences were not reported given the large sample sizes of different studies

[b] PR and PD are given between the countries with the highest and lowest prevalence of level 2 of the ATC classification. Dark gray indicates an ATC level 2 drug with a prevalence ratio (PR) > 2 and a prevalence difference (PD) ≥ 20 pediatric patients per 1000 per year.

© 2022 Taine M et al. *JAMA Network Open.*
